# Inhibition and induction of CYP enzymes in humans: an update

**DOI:** 10.1007/s00204-020-02936-7

**Published:** 2020-10-27

**Authors:** Jukka Hakkola, Janne Hukkanen, Miia Turpeinen, Olavi Pelkonen

**Affiliations:** 1 Research Unit of Biomedicine, Pharmacology and Toxicology, University of Oulu, POB 5000, 90014 Oulu, Finland; 2 Biocenter Oulu, University of Oulu, Oulu, Finland; 3 Medical Research Center Oulu, University of Oulu and Oulu University Hospital, Oulu, Finland; 4 Research Unit of Internal Medicine, Medical Research Center Oulu, University of Oulu and Oulu University Hospital, Oulu, Finland; 5 Administration Center, Medical Research Center Oulu, University of Oulu and Oulu University Hospital, Oulu, Finland

**Keywords:** Cytochrome P450, Inhibition, Induction, Drug–drug interaction, Herbal remedies, Environmental toxicants

## Abstract

The cytochrome P450 (CYP) enzyme family is the most important enzyme system catalyzing the phase 1 metabolism of pharmaceuticals and other xenobiotics such as herbal remedies and toxic compounds in the environment. The inhibition and induction of CYPs are major mechanisms causing pharmacokinetic drug–drug interactions. This review presents a comprehensive update on the inhibitors and inducers of the specific CYP enzymes in humans. The focus is on the more recent human in vitro and in vivo findings since the publication of our previous review on this topic in 2008. In addition to the general presentation of inhibitory drugs and inducers of human CYP enzymes by drugs, herbal remedies, and toxic compounds, an in-depth view on tyrosine-kinase inhibitors and antiretroviral HIV medications as victims and perpetrators of drug–drug interactions is provided as examples of the current trends in the field. Also, a concise overview of the mechanisms of CYP induction is presented to aid the understanding of the induction phenomena.

## Introduction

Inhibition and induction of cytochrome P450 (CYP) enzymes are central mechanisms, resulting in clinically significant drug–drug interactions (DDI). Today, characteristics and regulatory factors of various CYP enzymes have been elucidated to a considerable extent (Manikandan and Nagini 2018; Zanger and Schwab 2013). Detailed mechanisms of inhibition have been uncovered by studies on isolated or expressed enzymes and tissue fractions. Nuclear receptors as important xenobiotic-sensing transcription factors and as regulators of CYP induction have been elucidated (Wang et al. 2012).

Prediction on the basis of in vitro studies is now an integral part of early drug development (Lu and Di 2020) as well as of the medicines agency guidelines (EMA, FDA, and MHLW/PMDA). Computational models such as physiologically based pharmacokinetic models are now being used for quantitative prediction of in vivo interactions from in vitro experiments (Kato 2020; Min and Bae 2017), and these models are used extensively by drug developers before and during clinical trials. After preclinical studies, there is an ultimate need of human in vivo studies and observations on inhibition and induction. Obviously, such information is absolutely needed for clinical drug treatment to prevent possible adverse outcomes and ensure safety.

In addition to drugs, humans are exposed to a large number of other chemical substances through diet, use of cosmetics, in workplaces, by environmental pollutants, etc., and many of these chemicals are in vitro inhibitors or inducers of CYP enzymes but compared to pharmaceutics often poorly characterized. The risk posed by these chemicals is difficult or impossible to assess without reliable in vitro–in vivo extrapolation, which is only possible by having proven in vivo inhibitors or inducers (and non-effective substances) as reference items.

With these premises in mind, and pointing to the profound developments in drug research and regulation (see the guest editorial, Pelkonen et al., in this issue), we have collected and updated the information about human in vivo inhibitors and inducers, which would constitute a curated compilation for the use as a reference for other in-depth studies. The main focus is on data published after 2008, and in many instances, we point to our earlier review for references before 2008 (Pelkonen et al. 2008).

## Progress since 2008

We previously reviewed CYP inhibition and induction 12 years ago (Pelkonen et al. 2008). In 2008, we stated that, because multiplicity and variability of CYP enzymes are an important complicating factor in pharmacological and toxicological research and regulation, and predictive and pre-empting measures are a top priority, and thus, the development of predictive in vitro approaches is necessary and should be based on the firm background of basic research on the phenomena of inhibition and induction and their underlying mechanisms. Consequently, we focused on covering both inhibition and induction of CYP enzymes, always keeping in mind the basic mechanisms on which to build predictive and preventive in vitro approaches to be validated by in vivo studies. These principles still apply today. Nevertheless, since 2008, further progress has been made in the research of CYP inhibition and induction and the application of the knowledge. Furthermore, very important development has happened in the characteristics of new drugs.

## New pharmaceuticals since 2008

It is obvious that the spectrum of new drugs has changed since 2008 (see the guest editorial Pelkonen et al. in this issue and (de la Torre and Albericio 2020; Yu et al. 2019). Biological drugs, proteins, and peptides or oligonucleotides occupy nowadays a sizable share of new drugs (see Internet sites of major drug agencies: https://www.accessdata.fda.gov/scripts/cder/daf/; https://www.ema.europa.eu/en/medicines; https://www.pmda.go.jp/english/review-services/reviews/approved-information/drugs/0002.html) and their role in DDIs in general is supposed to be in the pharmacodynamics sphere; specifically, CYP-associated DDIs are not expected. Consequently, small-molecular new chemical entities represent a smaller contribution into the new drugs, and these are more thoroughly studied during the developmental phases with in vitro tools and during clinical trials with focus on specific enzymes and transporters depicted by the in vitro information. The efficiency of the in vitro and in vivo tools as formulated in guidance documents from major authorities (EMA 2012, FDA 2020, MHLW/PMDA 2018)^1^ is demonstrated by the fact that there have been no major surprises leading to drug withdrawals among novel drugs during the last 10–15 years. Advancements in the pharmacokinetic research include the recognition that many less-studied non-CYP enzymes and especially several transporters have emerged as interaction targets.

Shifts in approved drug classes have led to the situation that anticancer and antiviral (HIV) drugs are major molecules in CYP-associated DDIs. These shifts are probably behind the observation that CYP3A4 substrates form a majority of the drugs suspected or shown as causing CYP-associated interactions. The observation that there seem to be only a few inducers among newly approved drugs may be explained by the thrust in the development of small molecule drugs towards more potent and specific molecules. This has led to a relative decrease of clinical doses, which often are too small to cause a significant CYP induction.

## Tyrosine (protein) kinase inhibitors as an example of CYP-mediated DDIs

Tyrosine kinase inhibitors (TKIs) form a relatively novel class of (mainly) anticancer agents, which has been expanding tremendously over the last 2 decades. Because of their “precision” targets, TKIs offer a more effective and safer option in many cancers compared to the cytostatic agents. Because their pharmacodynamic targets are a diverse, even if functionally related, set of enzymes, it is not surprising that their chemical structures as well as their metabolism and general pharmacokinetic characteristics are rather variable. However, TKIs actually are well represented in DDI sections of reference books and reviews, especially regarding their metabolic features and transporter involvements [see, e.g., (Gay et al. 2017; Hussaarts et al. 2019; Jackson et al. 2018)]. In this section, the TKI-associated CYP-DDIs are presented as an example of current concerns of clinically important CYP interactions.

### Drugs selected

The drugs covered here include protein or tyrosine-kinase inhibitors (TKIs) approved by EMA and/or FDA until 2018. There are a number of TKIs that have been discarded in the last rounds of development, but this source of useful compounds remains largely untapped for the analysis of DDIs. However, a scan of literature and physician’s desk references demonstrate that many of the approved TKIs are predominantly CYP3A4 substrates and many of them display a potential to inhibit or induce CYP enzymes. Consequently, it is a good opportunity to look at various interaction characteristics of these TKIs for the purposes of this review. Some salient features are collected in Table 1.

**Table 1:**
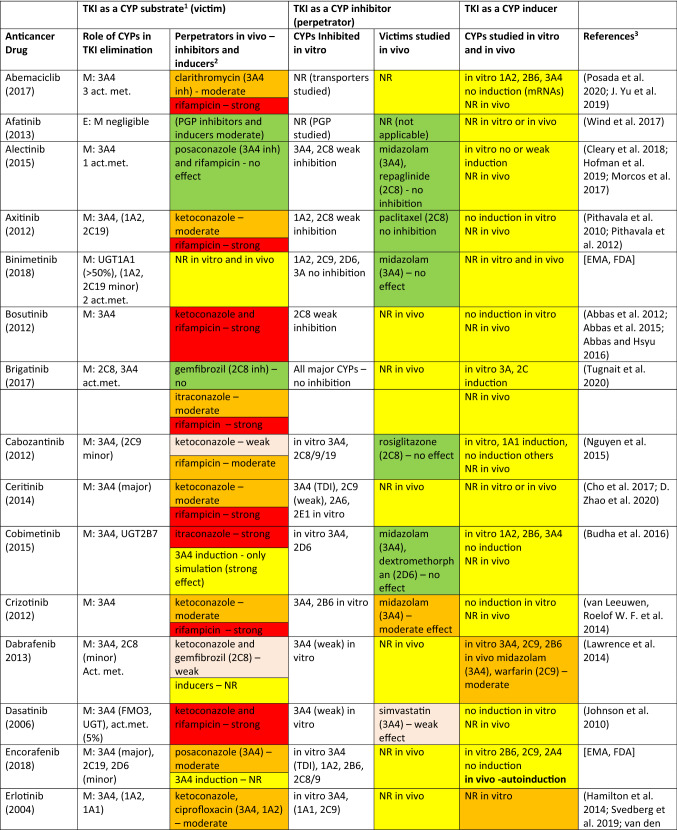

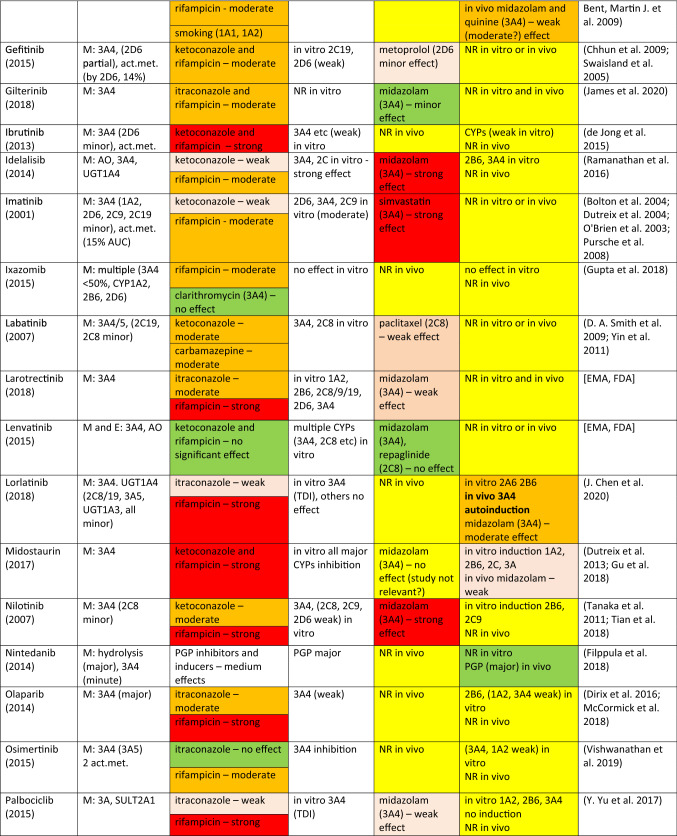

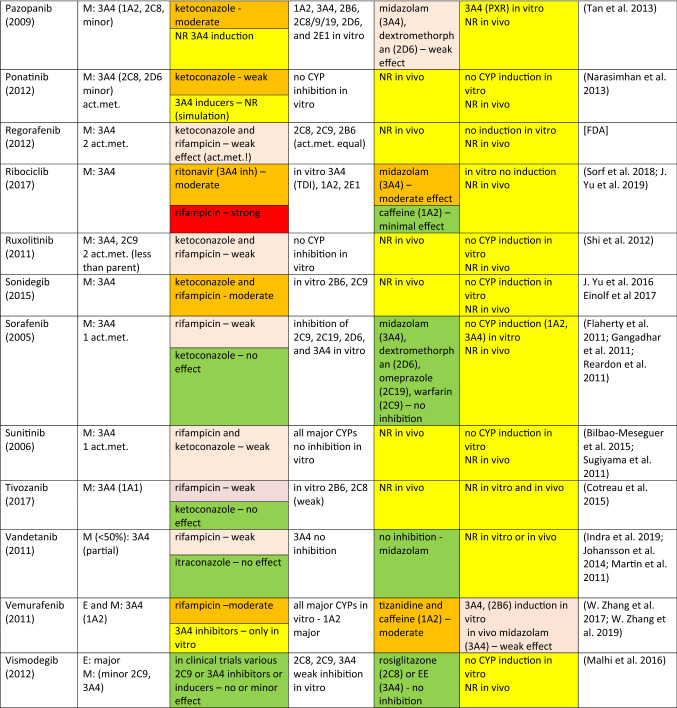
Tyrosine (protein) kinase inhibitor anticancer drugs as CYP substrates, inhibitors, and inducers

*Act.met.* active metabolite(s) (if reported or published), *PGP* P-glycoprotein, *NR* no results or not reported, *TDI* time-dependent inhibition

^1^E: excretion of a drug as an unchanged parent. M: metabolism—the extent and contributions of CYP isoforms’ other xenobiotic-metabolizing enzymes if known

^2^Usually, strong inducers (rifampicin) and inhibitors (ketoconazole, itraconazole) of CYP3A4 were studied. Other perpetrators are assigned with appropriate CYP enzyme. Color code: red, strong effect; orange, moderate effect; light brown, weak/minor effect; green, no (significant) effect; yellow, information in need

^3^Major sources drug monographs from FDA, EMA, and FIMEA; the latest uploaded documents were retrieved. Publications in general literature were sought and used for additional evidence for conclusions

### Key publications

An important element in research of TKIs is that the crucial development leading to authorization has occurred at the time when in vitro and in vivo studies for predicting and estimating CYP interactions have been refined to the extent that there has been a possibility for fact-based go/no-go decisions and that there are tools to estimate the contribution of particular CYP enzymes and their predictable interaction consequences. On the other hand, much of the available published material is of regulatory nature, i.e., drug monographs in national formularies, and thus detailed experimental and clinical results may not be available for open scrutiny. Thus, we have been mostly dependent on material that is not publicly peer-reviewed (naturally regulators have had access to original studies), but on the other hand, studies providing the basis for official drug monographs are expected to be of high quality. Furthermore, many of them have appeared in the public literature later on. Otherwise, publicly available studies are often rather sporadic regarding individual drugs, but, nevertheless, we have referred to them when they provide additional or confirmatory information.

### TKI as a victim drug

As can be seen in Table 1, a large majority of TKIs, 41 out of 43 drugs, is metabolized by CYP3A4/5 at least to a certain extent. Other CYP enzymes, such as CYP1A2, CYP2B6, CYP2C, and CYP2D6, contribute to the metabolism of some TKIs, but only binimetinib is metabolized to a small extent by CYP1A2 and CYP2C9 and not at all by CYP3A4/5. It is perhaps appropriate to note that the exact contribution of any single CYP is often rather difficult to quantitate precisely, but usually it is possible to state, whether CYP3A4 is responsible for a major or minor share of the metabolism. In vitro studies with human liver preparations or human hepatocytes are often crucial in this respect. In any case, it is not often possible to find in regulatory filings important parameters to describe enzyme kinetics, although some information may be found in the public literature.

The extent and relative isoform contribution of CYP-associated metabolism of individual TKIs is one of the crucial factors leading to clinically significant DDI potential. As the anticancer effect is of paramount interest for the developer of the compound, the clinician, and ultimately the patient, some risks of off-target effects including DDIs are accepted that would not be deemed acceptable when developing drugs for other less serious indications.

In DDI clinical studies, it is customary to use inhibitors and inducers which are known to have a strong effect. In most cases, rifampicin is used as an inducer and ketoconazole or itraconazole as an inhibitor. However, the strength of effect of a perpetrator is dependent on the metabolic characteristics of a victim, i.e., affinity to the principal enzyme, relative contribution of a specific enzyme to overall metabolism or PK behavior of a drug, and alternative enzymatic and excretory clearance routes. Consequently, the interaction outcome of a “strong” perpetrator may be strong, moderate, or weak, dependent on a specific victim. The intensity of inhibition or induction is defined by the FDA on the basis of the AUC change (FDA 2020).^2^ Strong, moderate, and weak inhibitors give rise to an increase in AUC of a victim at least fivefold, between two and fivefold, and 1.25- to 2-fold, respectively. For induction, corresponding AUC classes are an AUC decrease by > 80%, between 50 and 80% and between 20 and 50%. As stated above, even a “strong” inhibitor or inducer could result in strong, moderate, or weak effect, dependent on characteristics of a victim. Obviously, this classification provides only a rough yardstick for assessing the likelihood or clinical significance of an interaction and many other factors such as concentration–effect relationships of a victim may be more significant.

Regarding 43 TKI drugs in Table 1, the metabolism of 30 of them is strongly or moderately and seven weakly inhibited and/or induced by “strong” CYP3A4 perpetrators and only five are classified as having no CYP3A4-associated DDIs as victims. Among these “negatives”, CYP3A4 plays either a minor or no role in elimination: afatinib is excreted mainly unchanged, binimetinib is metabolized by hydrolysis, lenvatinib is predominantly excreted unchanged and metabolized by aldehyde oxidase, nintedanib is eliminated by P-glycoprotein, and vismodegib is eliminated only to a minor extent by CYPs. It is fair to conclude that a majority of clinically used TKIs are CYP3A4 substrates, although the contribution of CYP3A4 to the overall elimination may be decreased by other metabolic or transporter routes [see, e.g., (Fenner et al. 2009; Yu et al. 2017a, b, 2019)].

### TKIs as CYP inhibitors

Most TKIs in Table 1 have been screened for inhibitory potential using in vitro human liver microsomal assays consisting of major CYP activities from CYP1A2 to CYP3A4/5. In seven cases, no inhibition in vitro was detected, whereas for the rest of the drugs, the in vitro classifications ranged from “studied” to “some” or “weak inhibition”, and in a few cases even “moderate or strong inhibitory action”. However, on the basis of the published regulatory text, it is difficult to quantify “weak” or “strong” effect. Often, the regulatory text noted that inhibition was present or non-existent “at clinically relevant concentrations”. In certain cases, in vitro studies were followed by in vivo studies in which CYP-selective probe drugs were employed. For example, with respect to CYP3A4 substrates, inhibition was classified as strong for idelalisib–midazolam, imatinib–simvastatin and nilotinib–midazolam, moderate for crizotinib–midazolam, dasatinib–simvastatin, and ribociclib–midazolam, and weak for larotrectinib–midazolam, palbociclib–midazolam, and pazopanib–midazolam. Regarding CYP2D6 substrates, inhibition was classified as weak in two cases: gefitinib–metoprolol and pazopanib–dextromethorphan. Regarding CYP2C8, lapatinib inhibited weakly paclitaxel elimination, and with CYP1A2, vemurafenib inhibited moderately tizanidine and caffeine elimination. Altogether, it can be concluded that the cases CYP inhibition by TKIs, regarded worthy a warning in the regulatory desk reference, were rather few. However, occasionally, there were warnings that seemed to be based only on in vitro results and/or subsequent physiologically based pharmacokinetic (PBPK) simulations (Yu et al. 2019).

### TKIs as CYP inducers

According to the guidelines of major regulatory agencies, potential CYP induction should be studied in human-cultured hepatocytes in vitro or in an analogous cellular system. In most cases, appropriate studies have been performed and the outcome registered in the drug monograph. In 14 cases, no information on in vitro induction studies could be found (in Table 1, these are marked by NR, no results or not reported). No induction of the major inducible CYPs has been found in 14 cases and a clear response emerged in 10 cases (brigatinib, dabrafenib, ibrutinib, idelalisib, midostaurin, nilotinib, olaparib, osimertanib, pazopanib, and vemurafenib). In vivo studies were performed with 4 TKIs which resulted in a moderate induction with erlotinib–quinine or midazolam, and dabrafenib–midazolam or warfarin, and a weak induction with midostaurin–midazolam and vemurafenib–midazolam. Encorafenib was suspected of exhibiting autoinduction. However, regulatory texts are not always reliable regarding negative findings and it may well be that additional in vitro and in vivo studies have been performed but not reported. Based on this analysis, it can be concluded that TKIs do not often display clinically significant induction potency in humans in vivo.

### Active metabolites

At least 13 TKIs have at least one active metabolite. However, there may be several types of active metabolites regarding potential effects and outcomes. Several TKIs have pharmacodynamically active metabolites with a similar, although not necessarily equipotent, pharmacodynamic action as the parent. In some cases, a pharmacodynamically active metabolite may also have CYP-interaction potential. A special case is regorafenib, which has two CYP3A4-associated active metabolites with equal effect compared to the parent. This makes the assessment of interactions quite complex and uncertain. For example, although rifampicin exposure slightly decreased the AUC of the parent compound, it increased the AUC of one active metabolite by 2.6-fold. Thus, it is quite difficult to estimate the net pharmacodynamic effect.

Another mechanism is the so-called time-dependent inhibition (TDI), often due to the tight or irreversible binding of an active metabolite with the catalyzing enzyme leading to its inactivation (mechanism-based inhibition) or potentially due to formation of a more potent inhibitory metabolite. Both terms, TDI and mechanism-based inhibition, are used in this review. The evaluation of TDI would require appropriate in vitro studies, which were not usually available concerning TKIs. A recent review (Jackson et al. 2018) listed the following TKIs as potential candidates in this category: axitinib, bosutinib, dasatinib, imatinib, erlotinib, gefitinib, lapatinib, nilotinib, pazopanib, and sunitinib. However, company or authority data are not usually detailed enough in this respect, and more appropriate and detailed information is provided only rarely in published articles (Filppula et al. 2018; Kenny et al. 2012; Mao et al. 2016).

The generation of reactive metabolites has quite often been studied by drug companies developing the TKIs, since the reactive metabolites could potentially induce hepatotoxicity and form a threat for withdrawal during development or, worse, after the regulatory approval. Thus, at least in the following cases, reactive metabolites have been identified for clinically available tyrosine-kinase inhibitors: axitinib (Wang et al. 2020), dasatinib (Li et al. 2009), erlotinib (Li et al. 2009; Zhao et al. 2018), gefitinib (Li et al. 2009), imatinib (Li et al. 2014), lapatinib (Takakusa et al. 2011; Teng et al. 2010), ponatinib (Lin et al. 2017), and sunitinib (Amaya et al. 2018). It is, however, difficult to ascertain a specific reactive metabolite to cause a certain TDI, especially when the presence of a reactive metabolite has been deduced on the basis of trapping agents (Mao et al. 2016).

## Antiretroviral HIV drugs

The antiretroviral human immunodeficiency virus (HIV) drugs (Table 2) are of considerable interest for DDIs in research and therapy for two main reasons. First, the group contains two drugs (ritonavir and cobicistat) that are mainly used as pharmacokinetic enhancers, “boosters”, due to their strong and mechanism-based inhibitory action towards CYP3A4, the predominant enzyme metabolizing anti-HIV-protease inhibitors (Tseng et al. 2017). These boosters are rather rare examples of intentional, beneficial utilization of CYP-DDIs. The second reason is due to the frequent use of combinations of various antiviral drugs; up to four drugs in fixed combinations, although pharmacodynamic benefits are the major reasons to use such combinations.

**Table 2:** Antiretroviral HIV drugs as CYP substrates, inhibitors and inducers

Antiretroviral drug	As a CYP substrate		As a CYP inhibitor		As a CYP inducer	References^b^
	As a victim^a^	Perpetrators (effect assignments in parentheses)	Target enzymes	Victim drugs (effect assignments in parentheses)		
Pharmacokinetic enhancers (boosters)						
Cobicistat	E: > 80% M: 3A4, 2D6 (minor)	Strong 3A4 inducers (moderate)	3A4 (mechanism-based), 2D6 (weak)	Atorvastatin, rosuvastatin, etc.	No significant in vitro	Cattaneo et al. (2019), Sherman et al. (2015), Tseng et al. (2017)
Ritonavir	E: > 50% M: 3A4, 2D6 (minor)	Strong 3A4 inhibitors ketoconazole (minor) Strong 3A4 inducers rifampicin (moderate)	3A4 (mechanism-based), 2D6, 2C9	3A4-, 2D6- and 2C9-substrates variable effects	1A2, 2B6, 2C8, 2C9, 2C19 in vitro; in vivo minor or moderate effects	Cattaneo et al. (2019), Cooper et al. (2003), Tseng et al. (2017)
Protease inhibitors						
Atazanavir (+cobicistat)	M: 3A4	Strong 3A4 inducers rifampicin (strong) Efavirenz (moderate)	3A4 (mechanism-based), 2C8 (weak)	3A4 substrates (from weak to strong)	No effect in vitro or in vivo	Tseng et al. (2017)
Darunavir (+ritonavir)	M: 3A4, 2D6	3A4-inducers and inhibitors (variable observed or predicted effects)	3A4, 2D6	3A4 substrates (from weak to moderate)	2C9? warfarin	Tseng et al. (2017), Wagner et al. (2017)
Fosamprenavir (amprenavir) (+ritonavir)	M: 3A4	3A4-inducers and inhibitors (variable observed or predicted effects)	3A4	3A4 substrates (from weak to moderate)	3A4; in vivo effect minor or moderate	Justesen et al. (2003), Sale et al. (2002), Tran et al. (2002)
Lopinavir (+ritonavir)	M: 3A4	3A4-inducers and inhibitors (variable observed or predicted effects)	3A4	3A4 substrates (from weak to moderate)	3A4, in vivo effect minor at most	Wagner et al. (2017)
Nelfinavir	M: 3A4, 2C19	3A4-inducers and inhibitors (weak to moderate) 2C19-inhibitors (weak to moderate)	3A4	Midazolam (moderate)	In vitro 1A2, 2B6, 2C19 In vivo 1A2 (moderate), 2B6 (weak) and 2C9 (weak)	Kirby et al. (2011a, b)
Saquinavir (+ritonavir)	3A4	3A4-inducers and inhibitors (variable observed or predicted effects)	3A4	Midazolam (strong)	3A4, in vivo minor effect at most	Dickinson et al. (2008), Eagling et al. (2002)
Tipranavir (+ritonavir)	3A4	2B6 and 3A4-inducers and inhibitors (variable observed or predicted effects)	2D6	NA	3A4, 1A2, 2C19 combination in vivo moderate or strong effect	Tseng et al. (2017)
Integrase strand transfer inhibitors						
Bictegravir	M: 3A4, UGT1A1 (about equal)	3A4 inhibitors: voriconazole (weak), atanazavir (moderate) 3A4 inducers: rifabutin (moderate), rifampicin (strong)	No significant effects in vitro/in vivo	NA	No significant effects in vitro/in vivo	Gallant et al. (2017), Sax et al. (2017), Zhang et al. (2017)
Dolutegravir	E: ~ 50% M: UGT1A1; 3A4 (minor)	Strong 3A4 inducers: ritonavir, efavirenz, rifampicin (no significant effect)	No effect in vivo		No effect in vivo	Kandel and Walmsley (2015)
Elvitegravir	E: 95% M: 3A4 (minor)	Inducers; rifabutin, efavirenz, etc. (minor effect at most)	Minor effect in vitro at most		2C9?	Lee et al. (2012), Tseng et al. (2017)
Raltegravir	E: major M: UGT1A, no CYPs	No significant effects	No in vitro/in vivo		No in vitro/in vivo	Okeke and Hicks (2011)
Non-nucleoside reverse transcriptase inhibitors						
Doravirine	M: 3A4	Strong 3A4 inhibitors ritonavir, ketoconazole (moderate) Strong 3A4 inducers rifampicin (strong)	No in vitro/in vivo	NA	In vivo 3A4 (weak)	Khalilieh et al. (2019)
Efavirenz	M: 2B6 (primary), 2A6, 3A4	2B6 and 3A4-inducers and inhibitors (variable observed or predicted effects)	2C9, 2C19, 3A4	In vivo variable effects	3A4, 2B6 in vitro 2B6 autoinduction 2A6, 2B6, 2C19, 3A4 in vivo variable effects	Best and Goicoechea (2008), Marzolini et al. (2017), McDonagh et al. (2015), Metzger et al. (2019)
Etravirine	M: 3A4, 2C9, 2C19	Inhibitors and inducers variable effects	2C9, 2C19	In vitro variable effects	3A4	Havens et al. (2020)
Nevirapine	M: 3A4, 2B6	Rifampicin (moderate) Fluconazole (strong)	3A4, 2B6 (both weak)	Weak or no effects in vitro or in vivo	3A4, 2B6 In vivo autoinduction In vivo weak or moderate effect at most	Ena et al. (2012)
Rilpivirine	M: 3A4	Rifampicin (moderate) Ketoconazole (moderate)	3A4	No/minor effects in vivo at most	No in vitro/in vivo	Crauwels et al. (2013)
C–C chemokine receptor type 5						
Maraviroc	M: 3A4	Strong 3A4 inducers and inhibitors (strong)	3A4 (weak)	No significant inhibition in vitro or in vivo	No induction in vitro or in vivo	Abel et al. (2009)

^a^M, elimination by metabolism, E excretion as an unchanged drug

^b^Principal source for the information of this table is based on the AIDS Info: Panel on antiretroviral guidelines for adults and adolescents. Guidelines for the use of antiretroviral agents in adults and adolescents living with HIV. Department of Health and Human Services. 2020 [cited 2020 March 20]. Available from: https://aidsinfo.nih.gov/contentfiles/lvguidelines/adultandadolescentgl.pdf

The use of combinations makes it challenging to evaluate, especially in therapeutic situations, potential DDIs with other drug treatments of individual patients. The FDA or EMA-approved drug monographs contain extensive tabulated information about experimentally and/or clinically observed, or predicted DDIs, which often are difficult) to translate into clinically useful advice in actual patients. It is expected that in the future, DDI-predicting PBPK-models and artificial intelligence-based algorithms would aid clinical decisions [see, e.g., (Ryu et al. 2018; Varma et al. 2015)].

Cobicistat and ritonavir are especially employed in combination with HIV-protease inhibitors which are CYP3A4 substrates. CYP3A4-associated metabolism is very potently inhibited, because both boosters are mechanism-based inhibitors and block protease inhibitor metabolism and clearance almost completely thus extending drug exposure and the ensuing effect. They are also used in combination with other classes of HIV drugs, especially in fixed multidrug combinations containing protease inhibitors.

Pharmacokinetic interactions could also be based on processes involving transporters, e.g., P-glycoprotein. Many HIV drugs are ligands of various transporters and consequently interactions with other ligands may occur (Alam et al. 2016). This review will not cover transporter-mediated interactions as the focus is on CYP-DDIs.

Nucleoside reverse transcriptase inhibitors (abacavir, emtricitabine, lamivudine, tenofovir alafenamide, tenofovir disoproxil, and zidovudine) and the only fusion inhibitor (enfuvirtide) are devoid of CYP inhibition potential, because they are not metabolized by, or interacting with, CYP enzymes and most of them are renally eliminated. They are also not known to cause CYP induction.

## Herbal/botanical natural products interacting with drugs

Herbal and/or botanical (medicinal) products are used in the treatment of various diseases, often as a ‘self-treatment’ by the patient and many times unbeknownst to the treating physician (Paine and Roe 2018). From the drug-interaction point of view, a challenge is that herbal products are usually complex mixtures of constituents that can vary substantially in both content and concentration depending on the preparation and, furthermore, when isolated they can behave very differently (Kellogg et al. 2019; Paine et al. 2018; Sevior and Ahokas 2017). These problems are exaggerated by inadequacies of product regulation and standardization, thus leaving a physician without essential information and thus being at the mercy of very variable and often blatantly poor-quality literature (Pelkonen et al. 2014). Especially, there is a dearth of quality scientific data on potential herb–drug interactions for even widely used herbal medicines. In this review, interactions resulting in induction of CYP enzymes are detailed in Table 14. Regarding inhibitory interactions, only a few well-characterized examples (resveratrol, quercetin) have been included as ‘clinically significant’ perpetrators (see Table 4). According to literature reviews on herbal-associated CYP interactions [see, e.g., (Hermann and von Richter 2012; Izzo and Ernst 2009)], a large number of herbal preparations are interacting with CYP enzymes at the level of in vitro incubations, but there are variable and uncertain evidence on interactions in vivo. Also, major agency guidances pay little attention to these natural products; only EMA has a rather general entry in the interaction guidance, while FDA is treating herbal products as food supplements. The WHO document on herbal–drug interactions is under preparation and is expected shortly; it is hoped to set the stage for further scientific research and regulatory guidance to assess the clinical significance of herb–drug interactions.

## CYP substrates and inhibitors

### General

Data on substrates and inhibitors of major xenobiotic-metabolizing CYP enzymes are collected in Tables 3, 4, 5, 6, 7, 8, 9, 10 and 11. It is obvious that due to the vast literature, this survey cannot include all the possible substrates and inhibitors for CYP enzymes, instead certain restrictions had to be applied. Obviously, ‘the clinical significance’ is one of the overriding criterium, although it is very difficult to define. In this review, ‘the clinical significance’ means that the first-hand assessment of the drug, mostly on the basis of information in the regulatory dossier, has resulted in the inclusion of the drug in the list (see above the section on tyrosine-kinase inhibitors). However, ‘the clinical significance’ is dependent on many determinants including in vitro studies, clinical trials with reference substrates and inhibitors (these studies may be available at the time of approval), published non-regulatory studies and clinical experiences, etc. In the end, we have to admit that a certain measure of personal experience has been applied in the current review. Predominantly, only currently used drugs are listed, but some well-established, although withdrawn drugs are provided as reference. Also a few well-studied examples of in vitro substances are included because of their use as reference substrates or inhibitors.

**Table 3:** Substrates and inhibitors of CYP3A4/5 enzyme

Reference substrates recommended by major regulatory agencies^a^				
Drug	Reaction	Km (μM) in vitro (HLMs) (plasma conc)^b^	Specificity near Km	References
Midazolam in vitro, in vivo	1′-Hydroxylation/elimination	1–14 (0.8)	High	☺
Triazolam in vitro, in vivo	4-Hydroxylation/elimination	238–304 (0.06)	High	☺
Testosterone in vitro	6β-Hydroxylation	33–94 (na)	High	☺
Substrates potentially affected by strong CYP3A4 inhibitors^c^				
Highly selective/sensitive: alfentanil, alprazolam, aprepitant, atorvastatin, avanafil, budesonide, buspirone, colchicine, conivaptan, cyclosporin A, darifenacin, darunavir, dasatinib, dihydroergotamine (and ergotamine), docetaxel, dronedarone, ebastine, eletriptan, eliglustat, eplerenone, everolimus, felodipine, fentanyl, flibanserin, guanfacine, ibrutinib, indinavir, lomitapide, lovastatin, lurasidone, maraviroc, midazolam, naloxegol, nifedipine, nisoldipine, pimozide, quetiapine, quinidine, rilpivirine, rivaroxaban, saquinavir, sildenafil, simeprevir, simvastatin, sirolimus, sonidegib, tacrolimus, tadalafil, ticagrelor, tipranavir, tolvaptan, triazolam, vardenafil, and vincristine				
Additional protein tyrosine-kinase inhibitors, see Table 1 for details				

Reference inhibitors recommended by major regulatory agencies^a^				
Drug	Mode of inhibition	*K*_*i*_/IC_50_ (μM) in in vitro (plasma conc)^b^	CYP selectivity and other CYPs inhibited	References
Ketoconazole in vitro, in vivo	Competitive	0.0037–0.028 (2–6)	Moderate (2C, 1A2, 2D6)	☺
Itraconazole in vitro, in vivo	Competitive (metabolites)	0.013–0.27 (0.6–2.8)	High	☺ Yoshida et al. (2018)
Azamulin in vitro	Mechanism-based	0.03–0.24 (na)	High	Parmentier et al. (2017), Stresser et al. (2004)
Fluconazole	Competitive	5.4–13.1 (6–30)	Moderate (2C9, 2C19)	Niwa et al. (2005), Yoshida et al. (2018)
Troleandomycin in vitro	Mechanism-based	0.26	High	☺ Yadav et al. (2018)
Verapamil	Mechanism-based	2.3–2.9 (0.1–0.6)	High	☺
Ritonavir in vivo	Mechanism-based	0.019–0.17 (7–15)	Moderate (2C9)	☺
Clarithromycin in vivo	Mechanism-based (comp)	0.8 (5.5–10) (0.3–2.7)	High	☺
Erythromycin in vivo	Mechanism-based (comp)	1.0 (16–19) (1–8)	High	Akiyoshi et al. (2013), Kanamitsu et al. (2000)
Inhibitors of potential clinical significance				
Voriconazole	Mechanism-based	3.0 (4–17)	Poor (2B6, 2C9, 2C19)	Jeong et al. (2009a)
Posaconazole	Competitive	? (< 0.1?) (1)	High	Groll et al. (2017), Krishna et al. (2009)
Indinavir	Competitive	0.17–0.5 (> 0.16)	High	☺
Nelfinavir	Competitive	1–4.8 (> 1.4)	Moderate (CYP2D6)	☺
Saquinavir	Mechanism-based	0.65–2.99 (> 0.37)	High	☺
Diltiazem	Mechanism-based	2.2–5.0 (0.1–0.6)	High	☺
Telithromycin	Mechanism-based (competitive)	1.05 (3.65) (2.5)	High	Elsby et al. (2019)
Gestodene	Mechanism-based	46 (0.02)	High	☺ Palovaara et al. (2000)
**Ceritinib**	Mechanism-based	0.16–0.2 (0.9–2.7)	Moderate (2C9)	Zhao et al. (2020)
**Idelalisib**	Mechanism-based (metabolite)	5.1 (0.5–5)	High	Ramanathan et al. (2016)
Imatinib	Competitive?	8 (1–4)	Moderate	O’Brien et al. (2003)
**Lapatinib**	Mechanism-based	1.7	High (3A5: 37.6 uM)	Chan et al. (2012), Teng et al. (2010)
**Nilotinib**	Competitive	0.4–7 (2–3)	Moderate (2C8, 2C9, 2D6)	Tian et al. (2018)
**Osimertinib**	Mechanism-based competitive	2.5–5.1 (1.5–3)	Moderate (2C8)	Pilla Reddy et al. (2018), Vishwanathan et al. (2019)
**Stiripentol**	Competitive	80 (8–40)	Moderate (CYP1A2, 2D6)	Tran et al. (1997)
**Dronedarone**	Mechanism-based	0.87 (0.15–0.3)	Moderate (2J2)	Hong et al. (2016)
**Boceprevir**	Mechanism-based	6.1 (0.2–1.5)	High	Chu et al. (2013), Wilby et al. (2012)
**Telaprevir**	Mechanism-based	0.19–0.36 (3–4.5)	High	Chapron et al. (2015)
**Cobicistat**	Mechanism-based	0.032 (0.9)	Moderate	Hossain et al. (2017)
**Netupitant**	Competitive	1.9–5.7 (0.3–1)	Moderate (2C9)	Giuliano et al. (2012)
**Isavuconazole**	Competitive	0.62–1.93 (5.71)	Moderate (2C, 2D6)	Townsend et al. (2017), Yamazaki et al. (2017)
**Grapefruit juice**	Mechanism-based	Not applicable	Low? (multiple CYPs)	Bailey et al. (2013); Hanley et al. (2011)
Moderate inhibitors^c^ (regulatory documents): amprenavir, aprepitant, atazanavir, ciprofloxacin, crizotinib, darunavir/ritonavir, diltiazem, fosamprenavir, and gestodene				

*na* not available, *nk* not known

^☺^For older references, see (Pelkonen et al. 2008). Newer inhibitors, since 2008, have been indicated in bold

^a^Appropriate guidance documents of EMA (2012), FDA (2020), and MHLW/PMDA (2018) recommending the listed reference compounds for in vitro and in vivo studies. The use of two structurally unrelated CYP3A4/5 substrates for evaluation of in vitro CYP3A4/5 inhibition is recommended

^b^Km or *K*_*i*_/IC_50_ values were taken mostly from in vitro human microsomal incubations. Therapeutic (“control”) plasma concentrations were mainly taken from two compilations (Schulz et al. 2012, 2020) or the referenced publications listed

^c^The list is compiled from various published reviews, databases, and guidelines and drug labels of major drug agencies (EMA, FDA, MHLW/PMDA) as well as publicly available databases (Hoffmann et al. 2014; Preissner et al. 2010). Database address: http://bioinformatics.charite.de/transformer/

**Table 4:** Substrates and inhibitors of CYP1A2 enzyme

Reference substrates recommended by major regulatory agencies^a^				
Drug	Reaction/assay measurement	Km (μM) in vitro (plasma conc)^b^	Specificity near Km	References
Phenacetin in vitro probe (withdrawn)	*O*-De-ethylation	10–50 (na)	High	☺ Zhou et al. (2009)
Ethoxyresorufin in vitro probe (non-drug)	*O*-De-ethylation	0.11–0.23 (na)	Moderate (CYP1A1)	☺
Caffeine in vivo probe	*N*-Demethylation elimination rate (in vivo)^c^	200–500 (20–50)	High	☺ Thorn et al. (2012)
Theophylline in vivo probe	*N*-Demethylation elimination rate (in vivo)	280–1230 (10–30)	High	☺ Britz et al. (2019)
Tizanidine in vivo probe	Elimination rate (in vivo)	nk (0.6)	High	☺ (Granfors et al. (2005), Karjalainen et al. (2008)
Substrates potentially affected by strong CYP1A2 inhibitors^c^ (Faber et al. 2005; Wang and Zhou 2009)				
Sensitive/moderate: **agomelatine**, **alosteron**, clozapine, duloxetine, flutamide, frovatriptan, guanabenz, leflunomide, lidocaine, melatonin, mexiletine, mirtazapine, olanzapine, pirfenidone, propranolol, **ramelteon**, **ramosetron**, riluzole, **ropinirole**, ropivacaine, tacrine, **tasimelteon**, thalidomide, triamterene, zolmitriptan, zolpidem, and zileuton				

Reference inhibitors recommended by major regulatory agencies^a^				
Drug	Mode of inhibition	*K*_*i*_/IC50 (μM) in vitro (plasma conc)^b^	CYP selectivity (other CYPs inhibited)	References
α-Naphthoflavone in vitro (non-drug)	Competitive	0.01 (na)	Moderate (CYP1A1)	☺
Furafylline in vitro (withdrawn)	Mechanism-based	0.6–0.7 (nk)	High	☺
Enoxacin in vivo	Competitive	65–170 (3–12)	High	☺
Fluvoxamine in vivo	Competitive	0.12–0.24 (0.2–0.7)	Moderate (minor 2B6, 2C9, 2C19, 2D6)	☺
Inhibitors of potential clinical significance				
Amiodarone (metabolites)	Mechanism-based	0.46 (1.5–3)	Moderate (2D6, 3A4)	McDonald et al. (2015), Ohyama et al. (2000)
Ciprofloxacin	Competitive	90–290 (7.5–12)	High	☺ Granfors et al. (2004), Raaska and Neuvonen (2000)
Isoniazid	Competitive mechanism-based	56 (36–73)	Low (2C19, 3A4, 2A6)	Wen et al. (2002)
Mexiletine	Competitive	4.3–8.3 (3–11)	Moderate (1A1)	☺
Propafenone	Competitive	21 (1–6)	Moderate (2D6, 3A4)	☺ Dean (2012)
Thiabendazole	Mechanism-based	1.4 (na)	nk	Bapiro et al. (2005), Coulet et al. (1998), Thelingwani et al. (2009)
**Vemurafenib**	Competitive	~ 30 (100)	Moderate (2B6, 2C9, 3A4)	Zhang et al. (2017a, b)
Resveratrol (non-drug)	Competitive?	500 (na)	poor (1A1, 3A4)	Chang et al. (2001), Chun et al. (1999)
Moderate/weak inhibitors^c^: acyclovir, allopurinol, caffeine, cimetidine, daidzein, disulfiram, Echinacea, ethinylestradiol, famotidine, gestodene, norfloxacin, piperine, propafenone, propranolol, terbinafine, ticlopidine, verapamil, and zileuton				

*na* not available, *nk* not known

^☺^For older references, see (Pelkonen et al. 2008). Newer inhibitors, since 2008, have been indicated in bold

^a^Appropriate guidance documents of EMA (2012), FDA (2020), and MHLW/PMDA (2018) recommending the listed reference compounds for in vitro and in vivo studies. The use of two structurally unrelated CYP3A4/5 substrates for evaluation of in vitro CYP3A4/5 inhibition is recommended

^b^Km or *K*_*i*_/IC_50_ values were taken mostly from in vitro human microsomal incubations. Therapeutic (“control”) plasma concentrations were mainly taken from two compilations (Schulz et al. 2012, 2020) or the referenced publications listed

^c^The list is compiled from various published reviews, databases, and guidelines and drug labels of major drug agencies (EMA, FDA, and MHLW/PMDA) as well as publicly available databases (Hoffmann et al. 2014; Preissner et al. 2010). Database address: http://bioinformatics.charite.de/transformer/

**Table 5:** Substrates and inhibitors of CYP2B6 enzyme

Reference substrates recommended by major regulatory agencies^a^				
Drug	Reaction/assay measurement	Km (μM) in vitro (plasma conc)^b^	Specificity near Km	References
Bupropion (in vitro, in vivo)	Hydroxylation	89–130 (15–40)	High	☺
Efavirenz (in vitro, in vivo)	8-Hydroxylation	17–23 (3–10)	Moderate (CYP1A2, 3A4)	☺ Manosuthi et al. (2013)
Substrates potentially affected by strong CYP2B6 inhibitors^c^ (Hedrich et al. 2016)				
Highly/moderately sensitive: artemether, artemisinin, cyclophosphamide, diazepam, Ifosfamide, ketamine, mephenytoin, mephobarbital, methadone, nicotine, pethidine (meperidine), propofol, piclamilast, selegiline, and temazepam				

Reference inhibitors recommended by major regulatory agencies^a^				
Drug	Mode of inhibition	*K*_*i*_/IC50 (μM) in vitro (plasma conc)^b^	CYP selectivity and other CYPs inhibited	References
Ticlopidine (in vitro, in vivo)	Mechanism-based	0.2–0.8 (3–8)	Moderate (CYP1A2, 2C19, 2D6)	☺ Palacharla et al. (2018)
ThioTEPA (in vitro)	Mechanism-based	2.8–3.8 (3–20)	High	☺ Bae et al. (2013)
Sertraline (in vivo)	Competitive	3.2 (0.1–0.5)	Moderate	Hesse et al. (2000), Palacharla et al. (2018)
Phencyclidine (in vivo)	Mechanism-based	2 (0.1–1)	Moderate	Jushchyshyn et al. (2006), Walsky and Obach (2007)
Inhibitors of potential clinical significance				
**Canagliflozin**	Competitive	16 (0.6–3)	Poor (2E1, 3A4, 2C19, 2C9)	Yu et al. (2014)
Clopidogrel (pro-drug)	Mechanism-based	1.1 (0.02)	Moderate (2C19, 2C9)	☺ Backman et al. (2016), Wang et al. (2015)
17-α-Ethynylestradiol	Mechanism-based	0.8 (0.3 nM)	Moderate (1A2)	☺
**Sonidegib**	Competitive	0.045 (0.3–1)	Moderate (CYP2C9)	Yu et al. (2017a, b)
**Voriconazole**	Competitive	0.40 (5.7–11.5)	Poor (2C9, 2C19, 3A)	Jeong et al. (2009a, b)
Potential (moderate/weak) inhibitors^c^				

*na* not available, *nk* not known

^☺^For older references, see (Pelkonen et al. 2008). Newer inhibitors, since 2008, have been indicated in bold

^a^Appropriate guidance documents of EMA (2012), FDA (2020), and MHLW/PMDA (2018) recommending the listed reference compounds for in vitro and in vivo studies. The use of two structurally unrelated CYP3A4/5 substrates for evaluation of in vitro CYP3A4/5 inhibition is recommended

^b^Km or *K*_*i*_/IC_50_ values were taken mostly from in vitro human microsomal incubations. Therapeutic (“control”) plasma concentrations were mainly taken from two compilations (Schulz et al. 2012, 2020) or the referenced publications listed

^c^The list is compiled from various published reviews, databases, and guidelines and drug labels of major drug agencies (EMA, FDA, and MHLW/PMDA) as well as publicly available databases (Hoffmann et al. 2014; Preissner et al. 2010). Database address: http://bioinformatics.charite.de/transformer/

**Table 6:** Substrates and inhibitors of CYP2C8 enzyme

Reference substrates recommended by major regulatory agencies^a^				
Drug	Reaction/assay measurement	Km (μM) in HLMs (plasma conc)^b^	Specificity near Km	References
Repaglinide (in vivo)	Oxidation	24 (0.1–0.45)	Moderate (CYP3A4)	☺
Paclitaxel (in vitro)	6α-Hydroxylation	2.5–19 (0.3–0.8)	High	☺
Amodiaquine (in vitro)	*N*-De-ethylation	1.9–3.4 (0.15)	High	☺ Bohnert et al. (2016)
Substrates potentially affected by strong CYP2C8 inhibitors				
Highly selective: pioglitazone, rosiglitazone, and tazarotenic acid				
Moderately selective (other CYPs in parentheses): chloroquine (CYP3A4) and dasabuvir (3A4)				
Poorly selective (other CYPs in parentheses): amiodarone (CYP1A2, 2C19, 3A4)				

Reference inhibitors recommended by major regulatory agencies				
Drug	Mode of inhibition	*K*_*i*_/IC50 (μM) in vitro (plasma conc)^b^	CYP selectivity and other CYPs inhibited	References
Montelukast in vivo	Competitive	0.009–0.15 (0.05–0.5)	Moderate (CYP2C9, 3A4)	☺ Bohnert et al. (2016)
Quercetin in vivo (non-drug)	Competitive	1.1–1.6 (0.4)	Poor (CYP1A2, 2E1, 3A4)	☺
Phenelzine in vitro, in vivo	Mechanism-based	1.2 (0.1–1.5)		Kahma et al. (2019)
Clopidogrel in vitro, in vivo	Mechanism-based	na (0.02)	Moderate (CYP2C19, 2C9)	☺ Backman et al. (2016), Kahma et al. (2019), Tornio et al. (2014)
Gemfibrozil (glucuronide) in vitro, in vivo	Mechanism-based	52–75 (100)	High	☺ Kahma et al. (2019)
Inhibitors of potential clinical significance				
**Dabrafenib**	Competitive	8.2	Poor (2C9, 2C19, 3A4)	Lawrence et al. (2014)
**Deferasirox**	na	na (50)	Moderate (1A2. 3A4)	Pakkir Maideen et al. (2018), Skerjanec et al. (2010), Tanaka (2014)
Trimethoprim	Competitive	29–32 (4–9)	High	☺
**Teriflunomide**	na	na (100)	Moderate (1A2)	Cada et al. (2013)
**Vorapaxar**	Competitive?	0.86 (0.15)	Moderate (2C9)	Yu et al. (2016a, b)
**Belinostat**	na	100 (80)	Moderate (2C9)	Monograph
**Idelalisib**	Competitive?	13 (4)	Moderate (3A4, 2C9)	Yu et al. (2016a, b)
Potential and/or putative inhibitors:^c^ (Polasek et al. 2004) amiodarone, verapamil, nortriptyline, fluoxetine, and isoniazid. **tasimelteon**				

*na* not available, *nk* not known

^☺^For older references, see (Pelkonen et al. 2008). Newer inhibitors, since 2008, have been indicated in bold

^a^Appropriate guidance documents of EMA (2012), FDA (2020), and MHLW/PMDA (2018) recommending the listed reference compounds for in vitro and in vivo studies. The use of two structurally unrelated CYP3A4/5 substrates for evaluation of in vitro CYP3A4/5 inhibition is recommended

^b^Km or *K*_*i*_/IC_50_ values were taken mostly from in vitro human microsomal incubations. Therapeutic (“control”) plasma concentrations were mainly taken from two compilations (Schulz et al. 2012, Schulz et al. 2020) or the referenced publications listed

^c^The list is compiled from various published reviews, databases, and guidelines and drug labels of major drug agencies (EMA, FDA, and MHLW/PMDA) as well as publicly available databases (Hoffmann et al. 2014; Preissner et al. 2010). Database address: http://bioinformatics.charite.de/transformer/

**Table 7:** Substrates and inhibitors of CYP2C9 enzyme

Reference substrates recommended by major regulatory agencies^a^				
Drug	Reaction	Km (μM) in HLMs (plasma conc)^b^	Specificity near Km	References
S-warfarin in vitro, in vivo	7-Hydroxylation	3–4 (3–23)	High	☺
Diclofenac in vitro	4-Hydroxylation	2–22 (2–10)	High	☺
Tolbutamide in vivo	Hydroxylation	60–580 (150–340)	High	☺
Substrates potentially affected by strong CYP2C9 inhibitors:^c^ (Daly et al. 2017; Van Booven et al. 2010) bosentan, celecoxib, cyclophosphamide, flurbiprofen, fluvastatin, glibenclamide, glimepiride, glipizide, ibuprofen, indomethacin, irbesartan, lornoxicam, losartan, mefenamic acid, meloxicam, naproxen, nateglinide, phenytoin, tamoxiphen, and tenoxicam				

Reference inhibitors recommended by major regulatory agencies^a^				
Drug	Mode of inhibition	*K*_*i*_ (μM) in HLMs (plasma conc)^2^	CYP selectivity and other CYPs inhibited	References
Sulphaphenazole in vitro	Competitive	0.3 (300–500)	High	☺
Tienilic acid in vitro (withdrawn)	Mechanism-based	5 (150)	na	Hutzler et al. (2009)
Fluconazole in vivo	Mixed type	7–8 (6–30)	Poor (2C19, 3A4)	Back et al. (1988), Kunze et al. (1996)
Inhibitors of potential clinical significance				
Amiodarone	Non-competitive	95 (0.8–4)	Poor (2D6, 3A4)	Heimark et al. (1992), Ohyama et al. (2000)
**Ceritinib**	Mechanism-based	0.24 (0.9–2.7)	Moderate (3A4)	Zhao et al. (2020)
**Etravirine**	Competitive?	na (0.7–5)	Moderate (2C19)	Havens et al. (2020)
**Sonidegib**	Competitive	1.7 (0.3–1)	Moderate (3A4)	Yu et al. (2016a, b)
**Stiripentol**	Competitive	na (4–40)	Poor (1A2, 2D6, 3A4)	Tran et al. (1997)
**Vemurafenib**	Competitive	5.9 (100)	Poor (1A2, 2B6, 3A4)	(RW.ERROR—unable to find reference:doc:5ef341eae4b0f33707a95cec)
Moderate/weak inhibitors^c^: capecitabine, cotrimoxazole, fluvastatin, fluvoxamine, metronidazole, miconazole, oxandrolone, sulfinpyrazone, voriconazole, and zafirlukast (Wu et al. 2013)				

*na* not available, *nk* not known

^☺^For older references, see (Pelkonen et al. 2008). Newer inhibitors, since 2008, have been indicated in bold

^a^Appropriate guidance documents of EMA (2012), FDA (2020), and MHLW/PMDA (2018) recommending the listed reference compounds for in vitro and in vivo studies. The use of two structurally unrelated CYP3A4/5 substrates for evaluation of in vitro CYP3A4/5 inhibition is recommended

^b^Km or *K*_*i*_/IC_50_ values were taken mostly from in vitro human microsomal incubations. Therapeutic (“control”) plasma concentrations, either range or maximal, were mainly taken from two compilations (Schulz et al. 2012, 2020) or the referenced publications listed

^c^The list is compiled from various published reviews, databases, and guidelines and drug labels of major drug agencies (EMA, FDA, and MHLW/PDMA) as well as publicly available databases (Hoffmann et al. 2014; Preissner et al. 2010). Database address: http://bioinformatics.charite.de/transformer/

**Table 8:** Substrates and inhibitors of CYP2C19 enzyme

Reference substrates recommended by major regulatory agencies^a^				
Drug	Reaction	Km (μM) in HLMs (plasma conc)^c^	Specificity near Km	References
*S*-Mephenytoin (in vitro)	4′-Hydroxylation	23–169 (0.4–2)	High	☺
Omeprazole (in vivo)	5-Hydroxylation elimination	6–10 (0.2–10)	High	☺
Lanzoprazole (in vivo)	5-Hydroxylation elimination	15–17 (0.1–1)	Moderate (3A4)	☺
Substrates potentially affected by strong CYP2C19 inhibitors^c^				
Citalopram (2D6, 3A4), clobazam, clomipramine, diazepam (3A4), lansoprazole (3A4), pantoprazole (3A4), phenytoin, proguanil (3A4), propranolol, and rabeprazole (CYP3A4)				

Reference inhibitors recommended by major regulatory agencies^a^				
Drug	Mode of inhibition	*K*_*i*_/IC50 (μM) in vitro (plasma conc)^b^	CYP selectivity and other CYPs inhibited	References
-(−)-*N*-3-Benzyl-phenobarbital in vitro (non-drug)	Competitive	0.079–0.12 (na)	“Not specific”	Cai et al. (2004), Suzuki et al. (2002)
*S*-(+)-*N*-3-Benzyl-nirvanol in vitro (non-drug)	Competitive	0.2 (na)	“Not specific”	Suzuki et al. (2002)
Nootkatone in vitro (non-drug)	nk	0.5 (nk)	Poor (CYP2A6)	Tassaneeyakul et al. (2000)
Loratadine	Competitive	0.76 (0.05)	Poor (2D6, 3°4, 2E1)	Barecki et al. (2001), Ramanathan et al. (2018)
Ticlopidine	Mechanism-based	1.2 (3–8)	Poor (CYP2B6, 1°2, 2D6)	Ha-Duong et al. (2001), Ko et al. (2000), Turpeinen et al. (2006)
Inhibitors of potential clinical significance				
Omeprazole	Competitive	2–3 (0.2–10)	Moderate (2C9, 3A4)	Chiba et al. (1993), Funck-Brentano et al. (1997)
Fluvoxamine	Competitive	0.29 (0.13–0.53)	Moderate (1A2)	Iga (2016), Kong et al. (2014), Yasui-Furukori et al. (2004)
**Modafinil**	competitive	39 (6–15)	High	Robertson et al. (2000), Rowland et al. (2018)
Moderate/weak inhibitors^c^: Wu et al. (2013) Carbamazepine, cimetidine, esomeprazole, etravirine, felbamate, fluconazole, fluoxetine, ketoconazole, moclobemide, and voriconazole				

*na* not available, *nk* not known

^☺^For older references, see (Pelkonen et al. 2008). Newer inhibitors, since 2008, have been indicated in bold

^a^Appropriate guidance documents of EMA (2012), FDA (2020), and MHLW/PMDA (2018) recommending the listed reference compounds for in vitro and in vivo studies. The use of two structurally unrelated CYP3A4/5 substrates for evaluation of in vitro CYP3A4/5 inhibition is recommended

^b^Km or *K*_*i*_/IC_50_ values were taken mostly from in vitro human microsomal incubations. Therapeutic (“control”) plasma concentrations, either range or maximal, were mainly taken from two compilations (Schulz et al. 2012, 2020) or the referenced publications listed

^c^The list is compiled from various published reviews, databases, and guidelines and drug labels of major drug agencies (EMA, FDA, MHLW/PDMA) as well as publicly available databases(Hoffmann et al. 2014; Preissner et al. 2010). Database address: http://bioinformatics.charite.de/transformer/

**Table 9:** Substrates and inhibitors of CYP2D6 enzyme

Reference substrates recommended by major regulatory agencies^a^				
Drug	Reaction	Km (μM) in vitro (plasma conc)^b^	Specificity near Km	References
Bufuralol (withdrawn) in vitro	1′-Hydroxylation	3–30 (2)	High	☺
Dextromethorphan in vitro, in vivo	*O*-Demethylation	2.8–22 (0.5)	High	☺
Metoprolol in vivo	Elimination	7.4 (1.85)	High	Dean (2011), Berger et al. (2018)
Desipramine in vivo	2-Hydroxylation	10–15 (2.0)	High	☺
Nebivolol in vivo	Elimination	1.8 (0.05)	High	Hu et al. (2016), Lefebvre et al. (2007)
Substrates potentially affected by strong CYP2D6 inhibitors^c^				
Highly sensitive: atomoxetine, codeine, nortriptyline, perphenazine, tolterodine, and R-venlafaxine				
Moderately sensitive (other CYPs in parentheses): **eliglustat** (CYP3A4), encainide, imipramine, propafenone (CYP3A4), propranolol, thioridazine (CYP2C19, CYP3A4), tramadol (CYP3A4), trimipramine, and S-venlafaxine				

Reference inhibitors recommended by major regulatory agencies^a^				
Drug	Mode of inhibition	*K*_*i*_ (μM) in vitro (HLMs) (plasma conc)^b^	CYP selectivity and other CYPs inhibited	References
Quinidine in vitro, in vivo	Competitive	0.018–0.06 (6–15)	High	☺
Paroxetine in vitro, in vivo	Competitive	0.15 (0.01–0.2)	Moderate (2C9, 2C19)	☺
Fluoxetine in vivo	Competitive	0.6 (0.5–1.6)	Moderate (2C9, 2C19)	☺
**Mirabegron** in vivo	Mechanism-based	4.3 (0.01–0.2)	Moderate (CYP3A4)	Krauwinkel et al. (2014), Takusagawa et al. (2012)
Inhibitors of potential clinical significance				
Bupropion	Competitive	21 (15–40)	High	Reese et al. (2008), Sager et al. (2017)
Sertraline	Competitive	0.7 (0.02–0.5)	Moderate (2C9, 2C19)	☺
Terbinafine	Competitive	0.028–0.044 (0.03–0.1)	High	☺
**Stiripentol**	Competitive	(4–40)	Poor	Tran et al. (1997)
**Rolapitant**	Competitive	>7 (1)	High	Wang et al. (2017), Wang et al. (2018)
Potential inhibitors (mostly weak and/or putative)^c^: aprepitant, alogliptin, cobicistat, crizotinib, eliglustat, and panobinostat				

*na* not available, *nk* not known

^☺^For older references, see (Pelkonen et al. 2008). Newer inhibitors, since 2008, have been indicated in bold

^a^Appropriate guidance documents of EMA/EU (2012), FDA/USA (2020), and MHLW/PMDA (2018) recommending the listed reference compounds for in vitro and in vivo studies. The use of two structurally unrelated CYP3A4/5 substrates for evaluation of in vitro CYP3A4/5 inhibition is recommended

^b^Km or *K*_*i*_/IC_50_ values were taken mostly from in vitro human microsomal incubations. Therapeutic (“control”) plasma concentrations, either range or maximal, were mainly taken from two compilations (Schulz et al. 2012, 2020) or the referenced publications listed

^c^The list is compiled from various published reviews, databases, and guidelines and drug labels of major drug agencies (EMA, FDA, MHLW/PDMA) as well as publicly available databases (Hoffmann et al. 2014; Preissner et al. 2010). Database address: http://bioinformatics.charite.de/transformer/

**Table 10:** Substrates and inhibitors of CYP2A6 enzyme

Reference substrates (no recommendations by major regulatory agencies)				
Drug	Reaction/assay measurement	Km (μM) in in vitro HLMs (plasma conc)^a^	Specificity near Km	References
Nicotine in vitro (in vivo)^c^	*N*-1′-Oxidation (elimination)	65–95 (0.03–0.2)	High	☺
Coumarin in vitro (in vivo)^c^	7-Hydroxylation	0.2–2.4 (max. 5)	High	☺
Substrates potentially affected by strong CYP2A6 inhibitors^b^ (see (Tanner and Tyndale 2017) artemisinin, artesunate, caffeine, cotinine, letrozole, efavirenz, pilocarpine, tegafur, tyrosol, and valproic acid				

Reference inhibitors (no recommendations by major regulatory agencies)				
Drug	Mode of inhibition	*K*_*i*_ (μM) in HLMs (plasma conc)^a^	CYP selectivity and other CYPs inhibited	References
Tranylcypromine	Competitive	0.08–0.2 (0.4)	Moderate (2E1)	☺
Methoxsalen	Mechanism-based	0.2–0.8 (0.12–1)	Moderate (1A2)	☺
Inhibitors				
**Letrozole**	Competitive	4.6 (0.5)	Moderate (2C19)	Jeong et al. (2009a, b)
Pilocarpine	Competitive	1 (0.05)	High?	☺
*Trans*-cinnamic aldehyde (non-drug)	Mechanism-based	6.1 (nk)	High	Chan et al. (2016)
Tryptamine (non-drug)	Competitive	0.2 (nk)	Poor (CYP1A2)	☺

*na* not available, *nk* not known

^☺^For older references, see (Pelkonen et al. 2008). Newer inhibitors, since 2008, have been indicated in bold

^a^Km or *K*_*i*_/IC_50_ values were taken mostly from in vitro human microsomal incubations. Therapeutic (“control”) plasma concentrations, either range or maximal, were mainly taken from two compilations (Schulz et al. 2012, 2020) or the referenced publications listed

^b^The list is compiled from various published reviews, databases, and guidelines and drug labels of major drug agencies (EMA, FDA, and MHLW/PMDA) as well as publicly available databases (Hoffmann et al. 2014; Preissner et al. 2010). Database address: http://bioinformatics.charite.de/transformer/

^c^Nicotine and coumarin are used in various commodities, and could be used as probes also in vivo in small doses

**Table 11:** Substrates and inhibitors of CYP2E1 enzyme

Reference substrates (no recommendations by major regulatory agencies)				
Drug	Reaction	Km (μM) in vitro (HLMs) (plasma conc)^a^	Specificity near Km	References
Chlorzoxazone^a,b^	6-Hydroxylation	39–157 (170)	High	☺ Ernstgård et al. (2004)
*p*-Nitrophenol (non-drug)	3-Hydroxylation (nk)	24–30	High	☺ Collom et al. (2008)
Aniline (non-drug)	4-Hydroxylation	6–24	High	☺
Lauric acid (non-drug)	11-Hydroxylation	130	Moderate (CYP4A)	☺
Substrates potentially affected by strong CYP2E1 inhibitors^b^ acetaminophen (paracetamol), theophylline, enflurane, and halothane				

Reference inhibitors (no recommendations by major regulatory agencies)				
Drug	Mode of inhibition	*K*_*i*_/IC50 (μM) in vitro (plasma conc)^b^	CYP selectivity and other CYPs inhibited	References
4-Methylpyrazole	Competitive	2.0 (17–250)	High	Collom et al. (2008)
Diethyldithiocarbamate (DDC, non-drug)	Mechanism-based	5.3–34 (na)	Poor (1A2, 2A6, 2B6, 2C8, 3A4)	☺ Pratt-Hyatt et al. (2010)
Pyridine (non-drug)	Not known	0.4, 11.8 (na)	High	☺ Jones et al. (2011)
Disulfiram (in vivo)	Mechanism-based	Via DDC	Moderate (CYP2A6)	☺
Clomethiazole	Mechanism-based	1.0 (10)	Moderate (2A6)	☺ Stresser et al. (2016)
Diallyl sulfide (non-drug)	COMPETITIVE?	6.3–17.3 (na)	High?	☺ Rao et al. (2015)

*na* not available, *nk* not known

^☺^For older references, see (Pelkonen et al. 2008)

^a^ Km or *K*_*i*_/IC_50_ values were taken mostly from in vitro human microsomal incubations. Therapeutic (“control”) plasma concentrations, either range or maximal, were mainly taken from two compilations (Schulz et al. 2012, 2020) or the referenced publications listed

^b^The list is compiled from various published reviews, databases, and guidelines and drug labels of major drug agencies (EMA, FDA, MHLW/PMDA) as well as publicly available databases (Hoffmann et al. 2014; Preissner et al. 2010). Database address: http://bioinformatics.charite.de/transformer/

### Reference substrates and inhibitors

Reference substrates and inhibitors recommended by major regulatory agencies, FDA, EMA, and MHLW/PMDA, have been collected in the upper part of Tables 3, 4, 5, 6, 7, 8, 9, 10 and 11. The basic requirement is that the compound is metabolized totally or preferably by a single CYP enzyme, and this has been demonstrated in vitro and in vivo. In in vitro assay, the formation of the CYP-associated metabolite is followed, but in in vivo studies, often, the elimination of the parent is measured due to, e.g., further metabolism of a CYP-associated metabolite. Naturally, in the human in vivo studies, approved drugs have to be used, but the lists contain also a few substances which are either withdrawn drugs or experimental substances (e.g., azamulin). These are used only in in vitro tests to investigate basic in vitro interactions in connection with early drug development or in mechanistic studies later on.

### Sensitive substrates

In addition to reference substrates and inhibitors, appropriate lists of substrates and inhibitors of definitive clinical potential are compiled. Of potential substrates, only the so-called “strongly and/or moderately sensitive” substrates have been listed as extractions from reviews of individual CYP enzymes. Usually, sensitive substrates are metabolized almost completely or to a significant extent (> 25%) by the CYP enzyme concerned, so that the inhibition by a specific inhibitor will lead to a significant increase in the exposure to a substrate. However, there are a number of substrates which are actually metabolically activated by an enzyme and, consequently, the inhibition of metabolism leads to a pharmacodynamically reverse outcome and this is an important point to remember when assessing potential consequences of an interaction. However, perhaps, a more common situation is where pharmacologically active metabolites contribute to the action of the parent drug and the final outcome of the interaction may be more difficult to define.

### Clinically significant inhibitors

Among inhibitors, the listed substances contain mostly “strong” or at least “moderate” inhibitors for a given CYP enzyme. This implies a relatively strong affinity to an enzyme at concentrations achieved in clinical situations. For this reason, an inhibition constant or a corresponding measure (IC50, *K*_*i*_) and actual therapeutic concentration (if known) have been given in tables. Furthermore, mechanism of inhibition, most commonly competitive or mechanism-based inhibition, is of importance for the extent and length of inhibition.

The extent of inhibition is also heavily dependent on characteristics of a victim drug, its affinity to an enzyme, and a fraction of a victim metabolized by an enzyme. However, clinical situations could be much more complex. Consequently, quantitative measures of inhibitory potency are only guiding by nature, but may still suggest at least a significant possibility of inhibitory interaction in clinical drug use.

It should be kept in mind that the inhibition mechanisms may be very complex and may need extensive in-depth experiments to uncover the details of inhibition and the consequent in vitro and in vivo outcomes (Asaumi et al. 2018; Korzekwa et al. 2014; Lutz and Isoherranen 2012; Roberts et al. 2008; Varma et al. 2015). We have used a dichotomous expression of competitive vs mechanism-based inhibition, although the outcome of inhibition may be modified by more complex mechanisms.

It should also be stressed that the concentration of a drug interacting with the enzyme may be different from the plasma concentration, which is usually readily available from clinical trials and later monitoring activities. It has been suggested that the use of unbound cytosolic concentrations—as a proxy for total/unbound plasma concentrations—would improve the prediction of in vivo DDIs (Filppula et al. 2019). For practical reasons, we have listed the total plasma concentrations, not unbound concentrations, because there exists some uncertainty about which one is in better correlation with the drug concentration at the enzyme site. Also, it is not known whether there is a direct relation between unbound concentrations in plasma and cell cytosol. It has to be recognized that drugs bind to intracellular structures, mainly proteins and lipids, and the ensuing unbound concentration could be different from the unbound plasma concentration. A reliable method to measure the drug concentration at the effector site of an enzyme is needed.

Because the available literature on CYP inhibition is enormous, we have made use of our previous review (Pelkonen et al. 2008) as a collective reference to the older literature (Tables 3, 4, 5, 6, 7, 8, 9, 10, 11). In addition, we have referred to more recent papers if they have added significant new information. For many newer substances, publicly available regulatory dossiers have been a primary source of information, although they do not necessarily provide strictly quantitative information about DDIs.

### Substrates and inhibitors of individual CYPs

#### CYP3A4/CYP3A5

Table 3 presents a collection of compounds participating as substrates and/or inhibitors in clinically relevant CYP3A4-associated DDIs, which is by far the most important area of CYP-based interactions. The table lists also > 10 inhibitors (in bold), which have come to the market since our previous review in 2008 (Pelkonen et al. 2008).

On the basis of analyses of Yu et al. (2014, 2016a, b, 2017a, b, 2018, 2019) on FDA-approved drugs (close to 150 between 2013 and 2017), roughly 65% were substrates, 30% inhibitors and about 5% inducers of CYP3A. This is not to say that a similar portion should cause DDI consequences of clinical significance, because the establishment of clinical significance would require at least some in vivo trials and/or observations. Currently, the use of reference perpetrators (e.g., ketoconazole and rifampicin) or substrates (e.g., midazolam) is practically mandatory to aid the assessment of clinical significance.

Usually, it is not possible to indicate what would be a contribution of CYP3A5 for the DDI effect. However, if need be there are in vitro tools to study the CYP3A5 contribution into the metabolism or the effect of a studied drug (Guo et al. 2020; Lolodi et al. 2017). The most comprehensive literature on the role of CYP3A5 is available for tacrolimus, see (Birdwell et al. 2015; Chen and Prasad 2018).

#### CYP1A2

The list of substrates potentially affected by CYP1A2 inhibitors (Table 4) contains at least 13 “new” drugs [compared with the previous review in 2008 (Pelkonen et al. 2008)], whereas only one inhibitor of potential clinical significance, vemurafenib (see also Table 1), has appeared since 2008. Resveratrol has been added to the table as an example of an ingredient in a large number of consumable products, including red wine. However, it seems to be a moderate CYP1A2 inhibitor at the best.

#### CYP2B6

There are only three “new” drugs added into the list of inhibitors, canagliflozin, sonidegib, and voriconazole, and the first two are probably only moderate-to-weak inhibitors. The list of substrates potentially affected by strong CYP2B6 inhibitors contains almost exclusively “old” drugs.

#### CYP2C8

In addition of recommended substrates and inhibitors, Table 6 lists 6 ‘new’ inhibitors of CYP2C8. However, in the immediate analysis, some recently registered drugs, which were shown to be CYP2C8 inhibitors in in vitro studies, were difficult to classify. For example, according to the regulatory dossier studies, tasimelteon was shown to be a weak in vitro inhibitor of CYP2C8 (IC_50_ > 100 µM), whereas vorapaxar was a relatively potent in vitro inhibitor (IC_50_ 0.86 µM), but still both did not affect CYP2C8-associated rosiglitazone elimination in vivo [drug monographs, (Yu et al. 2016a, b)]. Consequently, tasimelteon is mentioned only in the group of putative inhibitors, waiting for additional in vivo investigations to classify more convincingly, whereas vorapaxar is listed in the category of inhibitors of potential clinical significance due to its low IC_50_ value as compared with the in vivo plasma concentration.

#### CYP2C9

The list of victim drugs of CYP2C9 (Table 7) is relatively long, altogether 20 substances. It reflects the importance of CYP2C9 in metabolizing clinically widely used drugs, practically all of which are “old” drugs and many of them used for 20–30 years. There are five “new” drugs as CYP2C9 inhibitors of potential clinical significance, three of them kinase inhibitors (ceritinib, sonidegib, and vemurafenib). The only “old” inhibitor is the widely used antiarrhythmic amiodarone, which is used in research projects as an example of a drug with a very long half-life, complex kinetics and multiple potential interactions (McDonald et al. 2015).

#### CYP2C19

Since the previous review (Pelkonen et al. 2008), only one “new” drug (modafinil) has been added in the list of inhibitors of potential clinical significance. Reference inhibitors recommended by major regulatory agencies are not specific for CYP2C19-mediated metabolism; however, they can be used together with other information such as data obtained from experiments done with recombinant enzyme systems.

#### CYP2D6

The classic polymorphic CYP enzyme was discovered decades ago, mainly based on debrisoquine hydroxylation studies. Debrisoquine, a classic probe drug [see (Pelkonen et al. 2008)], was withdrawn from clinical use a long time ago, and consequently from the lists of reference probe drugs. The current list of recommended reference inhibitors includes the only “new” drug, mirabegron (Table 9). In fact, there are not many “new” drugs listed in Table 9. One of the reasons may be the well-known problems related to CYP2D6 pharmacogenetics and drug–drug interactions, and likelihood of “killing” of molecules displaying CYP2D6 metabolism and/or inhibitory potency early in the drug development process.

#### CYP2A6

Since our review in 2008 (Pelkonen et al. 2008), only one drug (letrozole) has been added to the list of substrates or inhibitors (Table 10). Letrozole was added to the list of CYP2A6 inhibitors on the basis of an in vitro study (Jeong et al. 2009b); no clinical studies have been undertaken. Only 5 out of 102 FDA-approved drugs between 2013 and 2016 were at least partial substrates and/or inhibitors of CYP2A6 principally on the basis of in vitro experiments and none of them were considered as ‘clinically significant’ even potentially (Yu et al. 2018). Our own view over the years since 2007 (see the accompanying article, Pelkonen et al., this volume) is similar: although CYP2A6 was occasionally mentioned in drug labels as a target of in vitro inhibition (no quantitative information provided), no in vitro observations were translated into potentially clinical significance.

*CYP2E1* is another enzyme that has been only rarely observed to associate with clinically significant interactions (Table 11). According to our own experiences (Pelkonen et al., this volume) and those of Yu et al. (2014, 2016a, b, 2017a, b, 2018, 2019), CYP2E1 has been mentioned only rarely in drug monographs and there have been no ‘clinically significant’ interactions since 2008. This is also reflected in a lack of officially recommended reference compounds to study metabolism or inhibition associated with CYP2E1. However, it is known that CYP2E1 is of importance in the metabolism of several small-molecular xenobiotics and its role in biochemical consequences of heavy alcohol consumption should be duly noted.

## Mechanisms of CYP induction

### Xenobiotic-sensing receptors as mediators of CYP induction

The induction of drug metabolism has been known since 1950s and it was early on understood to have important consequences for the action of drugs. However, the mechanistic basis behind induction remained enigmatic for decades. Discovery of the xenobiotic-sensing receptors, aryl hydrocarbon receptor (AHR) at 1970s and pregnane X receptor (PXR) and constitutive androstane receptor (CAR) at 1990s, as the molecular mediators of the CYP induction was a major step forward in understanding the mechanisms of induction (Baes et al. 1994; Honkakoski et al. 1998; Kliewer et al. 1998; Poland et al. 1976).

The xenobiotic-sensing receptors are ligand-activated transcription factors belonging structurally either to the nuclear receptors or the basic-helix–loop–helix Per-Arnt-Sim (bHLH-PAS) proteins. Today, activation of these receptors and subsequent CYP induction can be studied with a number of in silico, in vitro, and cell-based methods enabling relatively good prediction of in vivo induction (Bernasconi et al. 2019; Kato 2020; Pelkonen et al. 2008). However, not all the compounds found to be activators in cell or other in vitro assays are actual in vivo activators because of pharmacokinetic or other factors. It has also become clear that AHR, PXR, and CAR not only control the elimination of xenobiotics, but regulate also many other endogenous functions and signaling pathways and their activation may be involved in many chronic diseases such as metabolic diseases and cancer (Hakkola et al. 2018).

### PXR and CAR, the xenobiotic-sensing nuclear receptors

PXR, systematic name NR1I2, and CAR, systematic name NR1I3, belong to the same subfamily of nuclear receptors. Their tissue expression profile is quite limited, and both are predominantly expressed in the liver, PXR also in the intestine (Wang et al. 2012). Low levels can be found in some other tissues. PXR and CAR ligand-binding sites have evolved to accommodate various foreign chemicals, and therefore, they play a major role in sensing of the chemical environment. The basis for their ligand promiscuity is large and flexible ligand-binding pockets that can accommodate a wide range of ligands with diverse structural and physicochemical properties (Buchman et al. 2018).

Especially, the PXR ligand-binding pocket is very large (1200–1600 Å^3^) and adaptable allowing a great number of compounds with different structures to bind and activate PXR, thus making PXR an ideal sensor for chemical environment (Buchman et al. 2018). The CAR ligand-binding pocket is smaller (~ 600 Å^3^) and less flexible than that of PXR and, therefore, apparently can accommodate a smaller number of chemicals (Buchman et al. 2018). However, also CAR can be activated with many different compounds. From the point of view of clinically important drug–drug interactions, PXR activation probably represents the most important induction mechanism. However, PXR and CAR also share many important pharmaceuticals as ligands.

While the DNA-binding domains of PXR and CAR are quite conserved across species, the ligand-binding domains differ significantly. Consequently, there are important species differences in the ligand preferences of these xenobiotic-sensing receptors hindering translation of in vivo results from the experimental animals to the humans (Blumberg et al. 1998; Lehmann et al. 1998). A classic example is rifampicin that induces efficiently the human PXR but poorly the mouse counterpart. Vice versa, PCN (pregnenolone-16α-carbonitrile) prefers the mouse PXR over the human PXR. Similarly, TCPOBOP (1,4-bis-[2-(3,5-dichloropyridyloxy)]benzene, 3,3′,5,5′-tetrachloro-1,4-bis(pyridyloxy)benzene) activates the mouse CAR, but not the human CAR, while CITCO (6-(4-Chlorophenyl)imidazo[2,1-b][1,3]thiazole-5-carbaldehyde-*O*-(3,4-dichlorobenzyl)oxime) is an agonist for the human CAR with little affinity to the mouse CAR (Chai et al. 2016). To overcome the problem of species differences in ligand preference, PXR and CAR-humanized mouse models have been developed (Scheer et al. 2008).

Aptly named as constitutive androstane receptor (or less frequently constitutively active receptor), CAR displays ligand-independent, constitutive transcriptional activity (Chai et al. 2016; Kobayashi et al. 2015). This has been especially evident in experiments utilizing exogenous expression of CAR in hepatic cell lines. In primary hepatocytes or in the liver in vivo, the constitutive activity may be limited by mainly cytoplasmic localization of the unliganded receptor as part of a multiprotein complex. Upon ligand binding, CAR dissociates from the chaperone proteins allowing translocation to nucleus. In addition to classical ligand binding, CAR may be activated indirectly. Phenobarbital is the prime example of an indirect CAR activator (Kobayashi et al. 2015). The mechanism of CAR activation by phenobarbital is complex and involves repression of epidermal growth factor (EGF) receptor (EGFR) signaling through the competitive inhibition of EGF–EGFR interaction. Subsequently, phosphorylation of receptor for activated C kinase 1 (RACK1) is reduced allowing RACK1 to interact with CAR and protein phosphatase 2A. This ternary interaction then enables CAR dephosphorylation and, consequently, translocation to nucleus (Kobayashi et al. 2015).

In response to ligand binding, both PXR and CAR transfer from the cytosol to the nucleus and form heterodimers with another nuclear receptor, retinoid X receptor (RXR). The heterodimer is then able to bind to the DNA elements including both direct and everted repeats of the sequence AGGTCA and its variants. The agonist-bound nuclear receptor activates transcription through coactivator recruitment modifying chromatin structure and engaging transcription initiation complex. In addition to this classical nuclear receptor function, PXR and CAR form also protein–protein interactions broadening the cellular functions under the control of these nuclear receptors (Oladimeji et al. 2016; Pavek 2016). This mode of action may be especially important for the gene repression by the receptors. Furthermore, the PXR and CAR function may be fine-tuned by phosphorylation status and other posttranslational modifications (Cui et al. 2016; Smutny et al. 2013; Staudinger et al. 2011).

PXR targets several CYP enzymes with major importance in drug metabolism including the most predominant drug-metabolizing CYP enzyme CYP3A4. Along with the CYP3A subfamily, PXR regulates many other important drug-metabolism CYPs. Chromatin immunoprecipitation sequencing (ChIP-Seq) analysis of PXR binding in HepG2 cells in response to rifampicin treatment detected rifampicin-induced regions close to *CYP2A6*, *CYP2B6*, *CYP2C8*, *CYP2C9*, *CYP2C19*, *CYP3A4,* and *CYP3A7* genes (Smith et al. 2014). In addition, several CYP genes with less-defined roles in drug metabolism and many phase 2 enzymes were found to interact with PXR (Smith et al. 2014).

CYP2B6 has been much studied as a classical CAR target gene, but the CAR target gene profile appears to be fairly overlapping with PXR (Kobayashi et al. 2015). No ChIP-Seq analysis revealing the CAR binding to human CYP genes has been published so far, although the human CAR interactome has been studied in a mouse model (Niu et al. 2018). Interestingly, this investigation showed that CAR targets several genes coding for other transcription factors including PXR and AHR introducing additional level of complexity to the induction mechanisms (Niu et al. 2018).

RXR functions as a binding partner for PXR and CAR as well as several other type 2 nuclear receptors. Although RXR is often regarded as a passive partner, RXR may also bind ligands such as 9-*cis* retinoic acid (de Almeida and Conda-Sheridan 2019) and it has been reported that RXR ligands may modulate function of the dimers formed by RXR and the xenobiotic-sensing receptors (Chen et al. 2010). It has also been reported that retinoids could induce CYP3A4 through RXR/VDR heterodimers and RXR homodimers (Wang et al. 2008).

### AHR

Aryl hydrocarbon receptor (AHR) belongs to the bHLH-PAS family of transcription factors (Nebert 2017). AHR is activated especially by toxins and environmental contaminants including the classical activator 2,3,7,8-tetrachlorodibenzo-*p*-dioxin (TCDD) and it has great toxicological significance (Kawajiri and Fujii-Kuriyama 2017). However, also some pharmaceutical ligands such as omeprazole activate AHR (Quattrochi and Tukey 1993). Many endogenous ligands have been identified for AHR including some originating from the microbiota (Bock 2019; Kawajiri and Fujii-Kuriyama 2017).

AHR is ubiquitously expressed in most tissues with high expression in placenta, lung, heart, pancreas, and liver (Dolwick et al. 1993). In absence of a ligand, AHR is sequestered to the cytosol in a complex with several proteins. Ligand binding-induced conformational change releases AHR from the chaperone proteins and allows translocation to the nucleus, where it heterodimerizes with another bHLH-PAS protein, aryl hydrocarbon receptor nuclear translocator (ARNT) (Kawajiri and Fujii-Kuriyama 2017; Nebert 2017). AHR/ARNT-dimer is then able to bind the so-called xenobiotic-response-elements (XRE) in the vicinity of the target genes to promote transcription. One of the target genes is aryl hydrocarbon receptor repressor (AHRR), which acts as a negative feedback mechanism (Bock 2019).

Among the CYPs, AHR mainly regulates the members of the CYP1 family, of which only CYP1A2 plays an important role in hepatic drug metabolism. In several extrahepatic tissues, AHR efficiently induces CYP1A1 and CYP1B1 (Bock 2019). In the other CYP families, AHR has been found to regulate some members in the CYP2 family including CYP2S1 (Saarikoski et al. 2005). In mouse, also Cyp2a5 is regulated by AHR, but no similar evidence exist for the human ortholog CYP2A6 (Arpiainen et al. 2005). AHR also regulates several phase 2 drug-metabolizing enzymes. In addition to drug metabolism, AHR plays important role in multiple physiological functions such as immunity, cell growth and differentiation, and prolonged activation may cause toxicity (Hakkola et al. 2018; Kawajiri and Fujii-Kuriyama 2017; Nebert 2017; Rothhammer and Quintana 2019).

### Other transcriptional mechanisms mediating CYP induction

In addition to the xenobiotic-sensing receptors, some other transcription factors have been shown to mediate induction of CYP enzymes in response to chemical exposure. Some classical steroid receptors have been shown to regulate CYP genes. In contrast to the xenobiotic-sensing nuclear receptors, these nuclear receptors are more restricted in ligand preference and act as homodimer. Accordingly, estradiol induces CYP2A6 directly through estrogen receptor α (ERα) binding to the 5′-flanking region of the gene (Higashi et al. 2007).

Glucocorticoids regulate CYP expression; however, the mechanisms are diverse. Some glucocorticoids such as dexamethasone are PXR ligands explaining the observed CYP induction. However, others like methylprednisolone activate poorly the human PXR (Shukla et al. 2011). In fact, glucocorticoid receptor (GR) activation induces expression of PXR and CAR that may explain in many cases the CYP induction by glucocorticoids (Pascussi et al. 2001, 2003). However, also direct GR-mediated regulation of the CYP2C and CYP3A genes has been reported (Chen et al. 2003; Ferguson et al. 2005; Gerbal-Chaloin et al. 2002; Hukkanen et al. 2003; Matsunaga et al. 2004). For the CYP3A genes, this has been shown in the lung and fetal liver, i.e., in the absence of PXR and CAR expression (Hukkanen et al. 2003; Matsunaga et al. 2004).

Nuclear factor-erythroid 2-related factor 2 (NRF2) (the official name: Nuclear factor-erythroid-derived 2-like 2, NFE2L2) is a transcription factor belonging to the cap-n-collar subfamily of basic region–leucine zipper-type transcription factors (Suzuki and Yamamoto 2015). NRF2 expression is controlled at the level of protein stability and under unstressed conditions NRF2 is targeted to proteasomal degradation by its interaction partner Kelch-like ECH-associated protein 1 (KEAP1). KEAP1 functions as a redox sensor and contains several highly reactive cysteines that, upon modification by electrophilic molecules, prevent it from targeting NRF2 for proteasomal degradation. Therefore, in response to oxidative stress, NFR2 is stabilized, accumulates to the nucleus, and forms heterodimers with small musculoaponeurotic fibrosarcoma oncogene homologue (sMAF) proteins. The NRF2/sMAF-dimer binds to the antioxidant response element (ARE) in the regulatory regions of the target genes (Cuadrado et al. 2019).

NRF2 pathway is activated in response to oxidative stress produced by many toxic compounds such as heavy metals like cadmium and lead (Abu-Bakar et al. 2013). NRF2 regulates multiple cell functions, among them antioxidative response and xenobiotic biotransformation (Cuadrado et al. 2019). However, within the xenobiotic metabolism machinery, NRF2 mainly targets phase 2 enzymes, and among the CYP enzymes, only a limited number of CYP2 genes are regulated by NRF2 (K. C. Wu et al. 2012). The best-characterized CYP target is the mouse gene Cyp2a5 (Abu-Bakar et al. 2007; Lämsä et al. 2010). Also the closely related human gene CYP2A6 is regulated by NRF2 (Abu-Bakar et al. 2013; Yokota et al. 2011). Interestingly, the AHR and NRF2 pathways crosstalk at multiple levels (Köhle and Bock 2007).

### Post-transcriptional regulation

Some CYPs are regulated at the post-transcriptional level. The most important example is CYP2E1. CYP2E1 protein has a short half-life and protein stabilization represents a major level of CYP2E1 regulation. The labile CYP2E1 protein is stabilized by xenobiotics such as ethanol, acetone, pyrazole, and isoniazid (Carroccio et al. 1994; Song et al. 1989). A few CYPs have been shown to be regulated by xenobiotics at the level of mRNA stability. mRNA stabilization has been shown convincingly for the mouse form Cyp2a5, which, in response to pyrazole treatment, is regulated by heterogeneous nuclear ribonucleoprotein A1 (hnRNP A1) binding to the 3′-untranslated region of the Cyp2a5 mRNA (Abu-Bakar et al. 2013). The human CYP2A6 appears to be regulated by a similar mechanism (Christian et al. 2004). During the recent years, many CYPs have been shown to be targeted by microRNAs that may also potentially mediate the post-transcriptional effects of chemical exposure (Yu et al. 2016a, b).

## The in vivo induction of human CYP enzymes with drugs, herbal medicines, and environmental chemicals

In the following section, we will present the current status on the knowledge of the human in vivo induction. The following tables present the medications (Table 12), environmental contaminants (Table 13), and the herbal remedies and nutritional exposures (Table 14) known to induce human CYP enzymes. Only human in vivo inducers are listed based on the following criteria: the compound induces a specific CYP enzyme as assessed by (1) the pharmacokinetics of an established CYP-specific probe, (2) the established CYP-specific metabolic pathway of an endogenous metabolite (such as 6β-hydroxycortisol and 4β-hydroxycholesterol for CYP3A4), or (3) tissue-level expression of a CYP enzyme mRNA or protein. Also, supporting in vitro mechanistic evidence was required for compounds with only one published report of in vivo induction. However, the mechanistic evidence was not required if the inducer was a structural analog of a well-established inducer (this pertains especially to various barbiturates). Supporting evidence was not required if at least two studies report the induction. For medications, only those in current clinical use are listed. For withdrawn pharmaceuticals, reader is advised to consult previously published reviews (Hukkanen 2012; Zanger and Schwab 2013). Only CYP enzymes in families 1–3 are covered here.

**Table 12:** Medications as in vivo inducers of human cytochrome P450 enzymes

Enzyme	Class of inducers	Inducing medication	Receptor(s) implicated	Tissues	References
CYP1A1	Proton pump inhibitors	Omeprazole	AHR	Duodenum	Buchthal et al. (1995), McDonnell et al. (1992)
CYP1A2	Antibiotics	Rifampicin	PXR indirectly?	Liver (phenotyping)	Backman et al. (2006), Robson et al. (1984), Wietholtz et al. (1995)
	Antiepileptics	Carbamazepine	CAR/PXR indirectly?	Liver (phenotyping and expression)	Lucas et al. (1998), Oscarson et al. (2006), Parker et al. (1998)
		Phenytoin	CAR/PXR indirectly?	Liver (phenotyping)	Miller et al. (1984), Wietholtz et al. (1989)
	Antiretrovirals	Nelfinavir	PXR indirectly?	Liver (phenotyping)	Kirby et al. (2011)
		Ritonavir	PXR indirectly?	Liver (phenotyping)	Hsu et al. (1998), Kirby et al. (2011), Penzak et al. (2002)
	Barbiturates	Pentobarbital	CAR and PXR indirectly?	Liver (phenotyping)	Dahlqvist et al. (1989)
		Phenobarbital	CAR and PXR indirectly?	Liver (phenotyping)	Landay et al. (1978), Saccar et al. (1985)
		Secobarbital	CAR and PXR indirectly?	Liver (phenotyping)	Paladino et al. (1983)
	Immunosuppressants	Teriflunomide	CAR?	Liver (phenotyping)	Aubagio summary of product characteristics^a^
	Proton pump inhibitors	Omeprazole	AHR	Liver (phenotyping and expression)	Diaz et al. (1990), Rost et al. (1994), Rost and Roots (1994)
CYP2A6	Antiepileptics	Carbamazepine	CAR/PXR	Liver (phenotyping and expression)	Oscarson et al. (2006), Williams et al. (2010)
	Antimalarials	Artemisinin	CAR/PXR	Liver (phenotyping)	Asimus et al. (2008)
	Antiretrovirals	Efavirenz	CAR/PXR	Liver (phenotyping)	Metzger et al. (2019)
	Barbiturates	Phenobarbital	CAR/PXR	Liver (expression)	Cashman et al. (1992), Kyerematen et al. (1990), Yamano et al. (1990)
	Estrogens	Ethinyl estradiol (of oral contraceptives)	ER	Liver (phenotyping)	Benowitz et al. (2006), Berlin et al. (2007), Sinues et al. (2008)
CYP2B6	Antibiotics	Rifampicin	PXR	Liver (phenotyping)	Chung et al. (2011), Loboz et al. (2006), Lopez-Cortes et al. (2002)
	Antiepileptics	Carbamazepine	CAR/PXR	Liver (phenotyping and expression)	Ji et al. (2008), Ketter et al. (1995, Oscarson et al. (2006)
		Phenytoin	CAR/PXR	Liver (phenotyping)	Slattery et al. (1996), Williams et al. (1999)
	Antimalarials	Arteether	CAR/PXR	Liver (phenotyping)	Elsherbiny et al. (2008)
		Artemether	CAR/PXR	Liver (phenotyping)	Elsherbiny et al. (2008)
		Artemisinin	CAR/PXR	Liver (phenotyping)	Elsherbiny et al. (2008), Simonsson et al. (2003), Zang et al. (2014)
		Artesunate	CAR/PXR	Liver (phenotyping)	Elsherbiny et al. (2008)
		Dihydroartemisinin	CAR/PXR	Liver (phenotyping)	Elsherbiny et al. (2008)
	Antipyretic analgesic	Metamizole	Unknown	Liver (phenotyping and expression)	Qin et al. (2012), Saussele et al. (2007)
	Antiretrovirals	Efavirenz	CAR/PXR	Liver (phenotyping), white blood cells	Kharasch et al. (2012), Meyer zu Schwabedissen et al. (2012), Ngaimisi et al. (2010), Robertson et al. (2008a, b)
		Nelfinavir	PXR	Liver (phenotyping)	Kirby et al. (2011a, b)
		Ritonavir	PXR	Liver (phenotyping)	Kharasch et al. (2008), Kirby et al. (2011a, b)
	Barbiturates	Phenobarbital	CAR/PXR	Liver (phenotyping)	Jao et al. (1972)
CYP2C8	Antibiotics	Rifampicin	PXR	Liver (phenotyping), small intestine enterocytes	Glaeser et al. (2005), Jaakkola et al. (2006), Niemi et al. (2000, 2004), Park et al. (2004)
		Flucloxacillin	PXR	Liver (phenotyping)	Du et al. (2013)
	Antiepileptics	Carbamazepine	CAR/PXR	Liver (expression)	Oscarson et al. (2006)
CYP2C9	Antiandrogens	Apalutamide	PXR?	Liver (phenotyping)	Duran et al. (2020)
		Enzalutamide	PXR	Liver (phenotyping)	Gibbons et al. (2015)
	Antibiotics	Dicloxacillin	PXR	Liver (phenotyping)	Stage et al. (2018)
		Nafcillin	PXR	Liver (phenotyping)	Kim et al. (2007), King et al. (2018)
		Rifabutin	PXR	Liver (phenotyping)	Lutz et al. (2018)
		Rifampicin	PXR	Liver (phenotyping), duodenum	Glaeser et al. (2005), O’Reilly (1974), Oscarson et al. (2007), Williamson et al. (1998), Zilly et al. (1975)
	Antiemetics	Aprepitant	PXR	Liver (phenotyping)	Depre et al. (2005), Shadle et al. (2004)
	Antiepileptics	Carbamazepine	CAR/PXR	Liver (phenotyping and expression)	Herman et al. (2006), Lai et al. (1992), Oscarson et al. (2006)
		Phenytoin	CAR/PXR	Liver (phenotyping)	Chetty et al. (1998), Dickinson et al. (1985)
	Antiretrovirals	Nelfinavir	PXR	Liver (phenotyping)	Kirby et al. (2011a, b)
		Ritonavir	PXR	Liver (phenotyping)	Kirby et al. (2011a, b), Lim et al. (2004), Yeh et al. (2006)
	Barbiturates	Pentobarbital	CAR/PXR?	Liver (phenotyping)	Yoshida et al. (1993)
		Phenobarbital	CAR/PXR	Liver (phenotyping)	Goldberg et al. (1996), Orme and Breckenridge (1976)
		Secobarbital	CAR/PXR?	Liver (phenotyping)	Breckenridge and Orme (1971), O’Reilly et al. (1980), Udall (1975)
	Endothelin receptor antagonists	Bosentan	PXR	Liver (phenotyping)	van Giersbergen et al. (2002), Weber et al. (1999a)
	Kinase inhibitor	Dabrafenib	PXR	Liver (phenotyping)	Suttle et al. (2015)
CYP2C19	Antiandrogens	Apalutamide	PXR?	Liver (phenotyping)	Duran et al. (2020)
		Enzalutamide	PXR	Liver (phenotyping)	Gibbons et al. (2015)
	Antibiotics	Dicloxacillin	PXR	Liver (phenotyping)	Stage et al. (2018)
		Rifampicin	PXR	Liver (phenotyping), duodenum	Feng et al. (1998), Oscarson et al. (2007), Zhou et al. (1990), Zilly et al. (1975)
	Antiepileptics	Carbamazepine	CAR/PXR	Liver (expression)	Oscarson et al. (2006)
		Phenytoin	CAR/PXR	Liver (phenotyping)	Richter et al. (1980)
	Antimalarials	Arteether	CAR/PXR	Liver (phenotyping)	Asimus et al. (2007), Elsherbiny et al. (2008)
		Artemether	CAR/PXR	Liver (phenotyping)	Elsherbiny et al. (2008)
		Artemisinin	CAR/PXR	Liver (phenotyping)	Asimus et al. (2007), Elsherbiny et al. (2008), Mihara et al. (1999), Svensson et al. (1998)
	Antiretrovirals	Efavirenz	CAR/PXR	Liver (phenotyping)	Michaud et al. (2012)
		Ritonavir (with lopinavir or tipranivir)	PXR	Liver (phenotyping)	Dumond et al. (2010), Yeh et al. (2006)
	Barbiturates	Pentobarbital	CAR/PXR?	Liver (phenotyping)	Heinemeyer et al. (1987)
		Phenobarbital	CAR/PXR	Liver (phenotyping and expression)	Lecamwasam et al. (1975), Richter et al. (1980)
CYP2E1	Antibiotics	Isoniazid	Stabilization	Liver (phenotyping), blood lymphocytes	Chien et al. (1997), Mazze et al. (1982), O’Shea et al. (1997), Walubo et al. (2005), Zand et al. 1993)
	Retinoid receptor modulators	All-*trans*-retinoic acid	RXR?	Liver (phenotyping)	Adedoyin et al. (1998)
CYP2S1	Retinoid receptor modulators	Topical all-*trans* retinoic acid	RXR?	Skin	Smith et al. (2003)
CYP3A4	Antiandrogens	Apalutamide	PXR?	Liver (phenotyping)	Duran et al. (2020)
		Enzalutamide	PXR	Liver (phenotyping)	Belderbos et al. (2018), Gibbons et al. (2015), Schwartzberg et al. (2017)
	Antibiotics	Dicloxacillin	PXR	Liver (phenotyping)	Stage et al. (2018)
		Flucloxacillin	PXR	Liver (phenotyping)	Fan et al. (2019)
		Nafcillin	PXR	Liver (phenotyping)	Lang et al. (2003)
		Rifabutin	PXR	Liver (phenotyping)	Barditch-Crovo et al. (1999), Perucca et al. (1988)
		Rifampicin	PXR	Liver (phenotyping and expression), duodenum	Greiner et al. (1999), Kolars et al. (1992), Marschall et al. (2005), McAllister et al. (1983), Ohnhaus and Park (1979), Perucca et al. (1988)
		Rifapentine	PXR	Liver (phenotyping)	Birmingham et al. (1978), Vital Durand et al. (1986)
	Antidiarrheals	Telotristat ethyl	PXR	Liver (phenotyping)	Yu et al. (2019)
	Antiemetics	Aprepitant	PXR	Liver (phenotyping)	Shadle et al. (2004)
	Antiepileptics	Carbamazepine	CAR/PXR	Liver (phenotyping, expression)	Crawford et al. (1990), Moreland et al. (1982), Oscarson et al. (2006)
		Phenytoin	CAR/PXR	Liver (phenotyping, expression)	Crawford et al. (1990), Thummel et al. (1994), Werk et al. (1964), Xu et al. (2006)
		Oxcarbazepine	PXR	Liver (phenotyping)	Andreasen et al. (2007), Klosterskov Jensen et al. (1992, Zaccara et al. (1993)
		Rufinamide	Unknown	Liver (phenotyping)	Perucca et al. (2008)
		Topiramate	PXR	Liver (phenotyping)	Rosenfeld et al. (1997)
	Antimalarials	Artemether	CAR/PXR	Liver (phenotyping)	Asimus et al. (2007)
		Artemisinin	CAR/PXR	Liver (phenotyping)	Asimus et al. (2007), Zang et al. (2014)
		Dihydroartemisinin	CAR/PXR	Liver (phenotyping)	Asimus et al. (2007)
	Antineoplastic agents	Vinblastine	CAR/PXR	Liver (phenotyping)	Smith et al. (2010)
	Antipyretic analgesic	Metamizole	Unknown	Liver (phenotyping and expression)	Caraco et al. (1999), Saussele et al. (2007)
	Antiretrovirals	Efavirenz	CAR/PXR	Liver (phenotyping)	Fellay et al. (2005), Mouly et al. (2002)
		Etravirine	PXR	Liver (phenotyping)	Kakuda et al. (2014), Scholler-Gyure et al. (2009)
		Fosamprenavir (and metabolite amprenavir)	CAR/PXR	Liver (phenotyping)	Justesen et al. (2003), Kashuba et al. (2005), Tran et al. (2002)
		Nevirapine	CAR/PXR	Liver (phenotyping)	Dailly et al. (2006), Mildvan et al. (2002), Solas et al. (2004)
		Ritonavir	PXR	Liver (phenotyping)	Hsu et al. (1997), Ouellet et al. (1998)
		Tipranavir	CAR/PXR	Liver (phenotyping)	Boehringer-Ingelheim (2005)
	Barbiturates	Pentobarbital	CAR/PXR?	Liver (phenotyping)	Berman and Green (1971), Schellens et al. (1989)
		Phenobarbital	CAR/PXR	Liver (phenotyping)	Back et al. (1980), Burstein and Klaiber (1965)
	Bile acid derivatives	Ursodeoxycholic acid	PXR	Liver (phenotyping)	Bodin et al. (2001), Marschall et al. (2005)
	Cystic fibrosis medications	Lumacaftor	PXR	Liver (phenotyping)	ORKAMBI summary of product characteristics^b^
	Endothelin receptor antagonists	Bosentan	PXR	Liver (phenotyping)	Dingemanse et al. (2003), Weber et al. (1999b)
	Glucocorticoids	Dexamethasone	GR/PXR	Liver (phenotyping)	McCune et al. (2000), Roberts et al. (2008), Watkins et al. (1989)
		Methylprednisolone	GR	Liver (phenotyping)	Kuypers et al. (2004), Villikka et al. (2001)
		Prednisolone	GR	Liver (phenotyping)	Press et al. (2010), van Duijnhoven et al. (2003)
		Prednisone	GR	Liver (phenotyping)	Anglicheau et al. (2003)
	Herpes virus medications	Amenamevir	Unknown	Liver (phenotyping)	Adeloye et al. (2018), Kusawake et al. (2017)
	Gout medications	Lesinurad	PXR	Liver (phenotyping)	Gillen et al. (2017)
	Retinoid receptor modulators	Alitretinoin (9-cis retinoic acid)	RXR	Liver (phenotyping)	Schmitt-Hoffmann et al. (2011)
		Bexarotene	RXR	Liver (phenotyping)	Padda et al. (2013), Wakelee et al. (2012)
	Steroidogenesis inhibitors	Mitotane	PXR	Liver (phenotyping)	Bledsoe et al. (1964), van Erp et al. (2011)
	Stimulants	Modafinil (and its *R*-enantiomer armodafinil)	Unknown	Liver (phenotyping)	Darwish et al. (2008), Robertson et al. (2002)
	Kinase inhibitors	Dabrafenib	PXR	Liver (phenotyping)	Lawrence et al. (2014)
		Erlotinib	PXR	Liver (phenotyping	Svedberg et al. (2019)
		Midostaurin	PXR	Liver (phenotyping)	Gu et al. (2018)
		Vemurafenib	PXR	Liver (phenotyping)	Zhang et al. (2017)
CYP3A5	Antibiotics	Rifampicin	PXR	Duodenum	Burk et al. (2004)
	Glucocorticoids	Topical clobetasol 17-propionate	GR	Skin	Smith et al. (2006)
CYP3A7 and CYP3A43	Antibiotics	Rifampicin	PXR	Duodenum	Oscarson et al. (2007)
	Antiepileptics	Carbamazepine	CAR/PXR	Liver (expression)	Oscarson et al. (2006)

Only medications currently in clinical use are listed

^a^https://www.accessdata.fda.gov/drugsatfda_docs/label/2020/202992s010lbl.pdf

^b^https://www.accessdata.fda.gov/drugsatfda_docs/label/2018/211358s000lbl.pdf

**Table 13:** Chemical toxicants and radiation as in vivo inducers of human cytochrome P450 enzymes

Enzyme	Class of inducers	Compound or exposure	Receptor(s) implicated	Tissues	References
CYP1A1	Dioxins	Various environmental exposures, and a case of massive TCDD poisoning	AHR	Skin	Fabbrocini et al. (2015), Saurat et al. (2012)
	PAHs	Charbroiled meat	AHR	Duodenum	Fontana et al. (1999)
		Smoking	AHR	Adipose tissue, lung, oral and pharyngeal mucosa, placenta, uroepithelium, fetal lung, fetal liver	Boyle et al. (2010), Chi et al. (2009), Dorrenhaus et al. (2007), Hukkanen et al. (2002), Huuskonen et al. (2008), McLemore et al. (1990), O’Shaughnessy et al. (2011), Pasanen et al. (1990), Tsai et al. (2018), Ullrich et al. (1997), Vyhlidal et al. (2013)
		Topical coal tar	AHR	Skin, hair follicles	Merk et al. (1987), Smith et al. (2006)
	Polychlorinated biphenyls	Consumption of contaminated rice oil	AHR	Placenta	Lucier et al. (1987)
	Radiation	Therapeutic ultraviolet-B radiation	AHR	Skin	Katiyar et al. (2000)
CYP1A2	Dioxins	Dioxins, mainly TCDD, from environmental and occupational exposures, an occupational accident, and a case of massive TCDD poisoning	AHR	Liver (phenotyping)	Abraham et al. (2002), Chernyak et al. (2016), Samer et al. (2020)
	Heterocyclic aromatic amines	Pan-fried meat	AHR	Liver (phenotyping)	Sinha et al. (1994)
	PAHs	Charbroiled meat	AHR	Liver (phenotyping)	Fontana et al. (1999), Kappas et al. (1978), Pantuck et al. (1976)
		Coffee	AHR	Liver (phenotyping)	Djordjevic et al. (2008), Horn et al. (1995)
		Smoking	AHR	Liver (phenotyping, expression in liver autopsy samples)	Baker et al. (2001), Hunt et al. (1976), Pantuck et al. (1972)
		Topical coal tar	AHR	Skin	Smith et al. (2006)
	Polybrominated and polychlorinated biphenyls	Consumption of contaminated fish and farm products	AHR	Liver (phenotyping)	Fitzgerald et al. (2005), Lambert et al. (1990)
CYP1B1	PAHs	Smoking	AHR	Adipose tissue, lung, oral mucosa, placenta, white blood cells, whole-blood cells, fetal lung	Boyle et al. (2010), Chi et al. (2009), Hukkanen et al. (2002), Huuskonen et al. (2008), Lampe et al. (2004), Tsai et al. (2018), van Leeuwen et al. (2007), Vyhlidal et al. (2013), Willey et al. (1997)
		Topical coal tar	AHR	Skin	Smith et al. (2006)
		Work in coke ovens and waste incinerators	AHR	White blood cells	Hanaoka et al. (2002), Hu et al. (2006)
	Radiation	Therapeutic ultraviolet-B radiation	AHR	Skin	Katiyar et al. (2000)
CYP2A6	Heavy metals	Cadmium	NRF2	Liver (phenotyping)	Satarug et al. (2004a, b)
CYP2E1	Benzene derivatives	Smoking (cigarette smoke contains both styrene and toluene, see below)	Stabilization?	Liver (phenotyping), bronchial epithelium	Benowitz et al. (2003), Oyama et al. (2007)
		Occupational exposure to styrene	Stabilization?	Blood lymphocytes, whole-blood cells	Prieto-Castello et al. (2010), Wongvijitsuk et al. (2011)
		Toluene	Stabilization?	Blood lymphocytes	Mendoza-Cantu et al. (2006)
CYP2S1	PAHs	Smoking	AHR	Bronchoalveolar macrophages	Thum et al. (2006)
		Topical coal tar	AHR	Skin	Smith et al. (2003)
	Radiation	Ultraviolet-B radiation	AHR	Skin	Smith et al. (2003)
CYP3A4	Organochlorine pesticides	Dichlorodiphenyltrichloroethane (DDT)	PXR	Liver (phenotyping)	Petersen et al. (2007), Poland et al. (1970)
		Endrin	PXR	Liver (phenotyping)	Jager (1970)

**Table 14:** Nutritional exposures and herbal remedies as in vivo inducers of human cytochrome P450 enzymes. Some of the studies have been performed with purified compounds in high doses for drug development purposes. Food contaminants and compounds formed during food preparation are listed in Table 13

Enzyme	Compound	Examples of sources	Receptor(s) implicated	Tissues	References
CYP1A2	Indole-3-carbinol	Cruciferous vegetables	AHR	Liver (phenotyping)	Pantuck et al. (1979), Reed et al. (2005)
	Resveratrol	Many plants including berries, grapes and peanuts, and red wine	AHR indirectly	Liver (phenotyping, studied only with a pharmacologic dose)	Chow et al. (2010)
CYP2A6	Genistein	Legumes such as soybeans	ER	Liver (phenotyping, studied only with a pharmacologic dose)	Chen et al. (2011)
	Sulforaphane	Cruciferous vegetables	NRF2	Liver (phenotyping)	Hakooz and Hamdan (2007)
	Quercetin	Tea, many vegetables, fruits, and berries	ER	Liver (phenotyping, studied only with a pharmacologic dose)	Chen et al. (2009)
CYP2B6	Baicalin	Baikal skullcap, an herbal remedy	CAR/PXR	Liver (phenotyping, studied only with a pharmacologic dose)	Fan et al. (2009)
	Hyperforin	St. John’s wort, an herbal remedy	PXR	Liver (phenotyping)	Lei et al. (2010)
	Sodium ferulate	Several herbal remedies such as *Angelica sinensis, Cimicifuga heracleifolia,* and *Lignsticum chuangxiong*	PXR	Liver (phenotyping, studied only with a pharmacologic dose)	Gao et al. (2013, 2012)
CYP2C9	Hyperforin	St. John’s wort	PXR	Liver (phenotyping)	Jiang et al. (2004, 2006)
CYP2C19	Baicalin	*Yin Zi Huang*, an herbal remedy with several herbs	CAR/PXR	Liver (phenotyping)	Fan et al. (2007)
	Hyperforin	St. John’s wort	PXR	Liver (phenotyping)	Wang et al. (2004a, b)
CYP2E1	Ethanol	Alcoholic drinks	Stabilization	Liver (phenotyping and expression), blood lymphocytes, esophagus, placenta	Girre et al. (1994), Millonig et al. (2011), Oneta et al. (2002), Perrot et al. (1989), Rasheed et al. (1997), Raucy et al. (1997, 1999), Takahashi et al. (1993), Tsutsumi et al. (1989)
	Unknown compound(s) in St. John’s wort	St. John’s wort	Unknown	Liver (phenotyping)	Gurley et al. (2002, 2005)
CYP3A4	Baicalin	*Yin Zi Huang*, an herbal remedy with several herbs	CAR/PXR	Liver (phenotyping)	Fan et al. (2007)
	Unknown compounds in *Echinacea purpurea*	*Echinacea purpurea*, an herbal remedy	PXR	Liver (phenotyping)	Gorski et al. (2004), Penzak et al. (2010)
	Ethanol	Alcoholic drinks	Stabilization	Liver (phenotyping and expression), duodenum (phenotyping)	Liangpunsakul et al. (2005), Luceri et al. (2001), Niemela et al. (2000), Rahmioglu et al. (2011)
	Genistein	Legumes, soybeans, coffee	PXR	Liver (phenotyping, studied only with a pharmacologic dose)	Xiao et al. (2012)
	Ginkgolide A and B	*Ginkgo biloba*, an herbal remedy	PXR	Liver (phenotyping)	Markowitz et al. (2003), Robertson et al. (2008b)
	Hyperforin	St. John’s wort	PXR	Liver (phenotyping), duodenum	Durr et al. (2000); Piscitelli et al. (2000); Roby et al. (2000)
	Quercetin	Many vegetables, fruits, and berries (also one of the flavonoids in *Ginkgo biloba*)	PXR	Liver (phenotyping, studied only with a pharmacologic dose)	Duan et al. (2012)
	Tanshinone IIA and cryptotanshinone	Danshen (*Salvia miltiorrhiza*), an herbal remedy	CAR/PXR	Liver (phenotyping), duodenum (phenotyping)	Qiu et al. (2010), Qiu et al. (2013), Zhou et al. (2018)

The search strategy included searching PubMed with the specific CYPs as keywords (e.g., CYP2B6 and [induction or inducer or induce]). Also searches with the specific probe compounds were performed (e.g., for CYP2B6 “bupropion and [induction or inducer or induce]”). The bibliographies of the publications were checked for additional articles. As the clinical and toxicological significance of the induction is often difficult to evaluate, the compounds are listed on the tables with no regard to the consequences or magnitudes of the induction. However, for CYP-inducing TKIs, Table 1 provides the estimates of potency. For the sake of brevity, the following paragraphs do not systematically repeat the data and the references given in Tables 12, 13 and 14.

The most important xenobiotic-activated receptor regulating the induction of enzymes in the CYP1 subfamily is AHR. Several environmental chemicals such as PAHs, dioxins, polychlorinated biphenyls, and heterocyclic aromatic amines induce CYP1A1, CYP1A2, and CYP1B1 enzymes via AHR (Tables 12, 13, 14). Human in vivo induction of CYP1A1 and CYP1B1 is difficult to study with phenotyping probes owing to their very low or non-existent hepatic expression and overlap with CYP1A2 substrates (Chang et al. 2003). However, their expression can be measured more easily as these enzymes are widely expressed in various extrahepatic tissues where tissue sampling is more convenient than with liver. Only one medication (omeprazole for CYP1A1 in duodenum and CYP1A2 in liver) (Buchthal et al. 1995; Diaz et al. 1990; McDonnell et al. 1992; Rost et al. 1994; Rost and Roots 1994) and one nutritional exposure (indole-3-carbinol present in cruciferous vegetables for hepatic CYP1A2) (Pantuck et al. 1979; Reed et al. 2005) are currently known to induce CYP1 enzymes via AHR-mediated pathways. PXR and CAR are not known to directly induce CYP1 enzymes but several CAR/PXR agonists do induce CYP1A2-related activities in vivo. It is quite likely that CAR/PXR agonists induce the expression of AHR and lead to the induction of CYP1 enzymes indirectly (Maglich et al. 2002; Oscarson et al. 2006). Recent evidence suggests that teriflunomide, an immunosuppressant, induces CYP1A2 activity as shown with caffeine phenotyping possibly via phenobarbital-like indirect CAR activation (Carazo et al. 2018).^3^

CYP2A6 is induced in humans in vivo by CAR, PXR, ERα, and NRF2 agonists (Tables 12, 13, 14). The regulation of CYP2A6 by ERα and NRF2 sets it apart as no other CYP enzyme is known to be regulated in vivo by these transcription factors. CYP2A6 is induced through ERα by phytoestrogens such as genistein (in legumes such as soybeans)(Y. Chen et al. 2011; Mazur 1998) and quercetin (in tea, vegetables, fruits, and berries) (Chen et al. 2009; Chun et al. 2012) as well as ethinyl estradiol of oral contraceptives (Benowitz et al. 2006; Berlin et al. 2007; Sinues et al. 2008). Exposure to cadmium measured as urine cadmium excretion is associated with CYP2A6 activity probed with coumarin 7-hydroxylation but only in non-smokers (Satarug et al. 2004a, b). In smokers, CYP2A6 activity is known to be reduced (inhibition) by an unknown mechanism (Hukkanen et al. 2005) and as smoking is also an important source of cadmium (induction), it is not surprising that smoking can confound the association between cadmium exposure and CYP2A6 activity. The effect of cadmium on CYP2A6 is most likely mediated by NRF2 as is the induction caused by sulforaphane present in cruciferous vegetables (Abu-Bakar et al. 2004; Yokota et al. 2011). All medications known to induce CYP2A6 are combined CAR/PXR activators and it is not known which nuclear receptor is more important for CYP2A6 induction in vivo as there is some evidence for the involvement of both (Itoh et al. 2006). Rifampicin treatment for 6 days had no effect on CYP2A6 activity measured as coumarin hydroxylation (Rautio et al. 1994) arguing against the role of PXR in the in vivo regulation.

Several medications with PXR and combined CAR/PXR-activating properties induce CYP2B6 (Table 12). The mechanism mediating the effect of metamizole, an antipyretic analgesic with spasmolytic properties, on the induction of CYP2B6 is currently unknown (Qin et al. 2012; Saussele et al. 2007). It is not acting as a direct ligand of PXR or CAR and an indirect phenobarbital-like mechanism has been suggested (Qin et al. 2012; Saussele et al. 2007). No environmental toxicant has been shown to induce CYP2B6 in vivo, but constituents of herbal remedies such as baicalin (CAR/PXR), hyperforin (PXR) of St. John’s wort, and sodium ferulate (PXR) induce CYP2B6 (Fan et al. 2009; Gao et al. 2012, 2013; Lei et al. 2010) (Table 14). The effects of baicalin and sodium ferulate on CYP2B6 were demonstrated only as purified compounds in high doses. Thus, it is not known if dosing as herbal preparations containing *Angelica sinensis*, *Cimicifuga heracleifolia*, or *Lignsticum chuangxiong* (sodium ferulate) or Baikal skullcap (*Scutellaria baicalensis*) (baicalin) induce CYP2B6.

The induction of CYP2C8 has been demonstrated only with a few CAR or PXR-activating pharmaceuticals (Table 12). No environmental chemicals or constituents of herbal remedies are known to induce CYP2C8 in vivo in humans. Similarly, CYP2C9 is not known to be induced by environmental toxicants and only one herbal preparation, St. John’s wort, induces CYP2C9-related activities in vivo (Jiang et al. 2004, 2006). However, a multitude of medications (PXR agonists and combined CAR/PXR activators) induce CYP2C9 (Table 12). CYP2C19 induction has been demonstrated with baicalin-containing Chinese multicomponent herbal preparation *Yin Zhi Huang* and hyperforin-containing St. John’s wort (Fan et al. 2007; Wang et al. 2004a, b) (Table 14), while no environmental chemical is known to induce CYP2C19. Several medications with PXR and CAR/PXR-activating properties induce CYP2C19 (Table 12).

The induction of CYP2E1 is regulated unlike any other CYP enzyme. The stabilization of mRNA and protein by inducing compounds, many of which are also CYP2E1 substrates, is the main mechanism of induction (Cederbaum 2006). Benzene derivatives such as styrene and toluene encountered by workers in print and plastic industries are known to induce CYP2E1 (Mendoza-Cantu et al. 2006; Prieto-Castello et al. 2010; Wongvijitsuk et al. 2011) and the same compounds may also be responsible for the CYP2E1 induction detected in tobacco smokers (Benowitz et al. 2003; Oyama et al. 2007) (Table 13). The most well-known toxicant inducing CYP2E1 is ethanol (Table 14). Two medications have been demonstrated to induce CYP2E1, namely isoniazid (stabilization) and oral all-*trans* retinoic acid with RXR agonism as the most likely mode of induction (Gyamfi et al. 2006) (Table 12). St John’s wort induces CYP2E1 in long-term administration (28 days), but the mechanism is unknown (Gurley et al. 2002, 2005). It is not known if the well-established PXR agonist hyperforin is involved or if some other St. John’s wort ingredient is responsible for the induction of CYP2E1.

CYP2S1 is induced in skin and bronchoalveolar macrophages with exposures containing AHR agonists such smoking and topical coal tar (G. Smith et al. 2003; Thum et al. 2006) (Table 13). Ultraviolet-B (UVB) radiation has been demonstrated to induce CYP2S1 in skin with AHR-mediated mechanism which is also involved in the induction of cutaneous CYP1A1 and CYP1B1 by UVB (Katiyar et al. 2000; Smith et al. 2003). UVB exposure leads to the formation of 6-formylindolo[3,2-b]carbazole, a tryptophan photoproduct and an endogenous AHR ligand (Fritsche et al. 2007). The only medication known to induce CYP2S1 expression is topical all-*trans* retinoic acid, possibly via RXR (McNeilly et al. 2012).

As CYP3A4 is involved in the metabolism of approximately 50% of all marketed medications (Zhou 2008), its induction is of special importance. There are also numerous pharmaceutical CYP3A4 inducers leading to increased risk of drug–drug interactions (Table 12). CAR, GR, and PXR are known to mediate the induction. The mechanism of induction is unknown for antiepileptic rufinamide, stimulants modafinil and its *R*-enantiomer armodafinil, antiherpetic medication amenamevir, and metamizole (Table 12). Also RXR agonists alitretinoin (9-cis retinoic acid) and bexarotene are known to induce CYP3A4-related activities in phenotyping studies (Padda et al. 2013; Schmitt-Hoffmann et al. 2011; Wakelee et al. 2012).

In addition to CYP3A4-inducing medications, quite many herbal remedies and food ingredients induce CYP3A4 (Table 14). Also the occupational and environmental exposure to organochlorine pesticides dichlorodiphenyltrichloroethane (DDT) and endrin is associated with the induction of CYP3A4 as measured with urinary 6β-hydroxycortisol (Petersen et al. 2007; Poland et al. 1970) (Table 13). One often neglected CYP3A4 inducer is ethanol. Chronic alcoholics had a higher ratio of urine 6β-hydroxycortisol/cortisol compared with healthy volunteers (Luceri et al. 2001). Also oral bioavailability of midazolam was significantly lower in subjects with moderate alcohol consumption in comparison with abstaining controls suggesting intestinal CYP3A4 induction (Liangpunsakul et al. 2005). In a twin study, alcohol consumption was significantly associated with greater St. John’s wort-induced CYP3A4 activity as assessed with quinine phenotyping (Rahmioglu et al. 2011). There are also indications that CYP3A4 protein could be induced in liver of the alcoholics with liver disease (Niemela et al. 2000).

The evaluation of induction phenomena of CYP3A enzymes is complicated by the closely related CYP3A5 enzyme. CYP3A4 and CYP3A5 have widely overlapping substrate specificities and their regulation shares certain features such as crucial role of PXR and CAR (Burk et al. 2004). A notable difference is the extensive influence of genetics on CYP3A5 expression. The CYP3A5*3 allele with severely decreased enzymatic activity is more common than the CYP3A5*1 allele (CYP3A5*3 allele frequency is ~ 90% in Caucasians and 50% in African–Americans) (Lamba et al. 2002). Thus, most Caucasians do not have a functional CYP3A5 enzyme. The phenotyping studies performed with probes metabolized by CYP3A4 and CYP3A5 are classified here as showing only CYP3A4 induction if there are no enzyme-specific data on CYP3A5 induction. It is conceivable that many of the CYP3A4 inducers are also CYP3A5 inducers in those patients carrying one or two functional CYP3A5*1 alleles. There are only a few known CYP3A5 in vivo inducers. Rifampicin induced duodenal CYP3A5 mRNA in the subjects carrying a CYP3A5*1 allele, while no induction was detected in CYP3A5*3/*3 subjects (Burk et al. 2004). Topical administration of the glucocorticoid clobetasol 17-propionate induced cutaneous CYP3A5 mRNA (Smith et al. 2006).

The induction of minor CYP3A forms has also been demonstrated. The use of carbamazepine is associated with the increased expression of hepatic CYP3A7 and CYP3A43 mRNA (Oscarson et al. 2006). Rifampicin induces intestinal CYP3A7 and CYP3A43 mRNA in healthy volunteers (Oscarson et al. 2007) (Table 12).

## Consequences and relevance of CYP induction

The induction of CYP enzymes as a cause of DDIs, as distinct from the enzyme inhibition, is unique as the induction becomes apparent more slowly and it takes more time for the induction to abate. This is caused by the delay due to the synthesis of new enzymes when the inducer is introduced, and then for the additional enzymes to degrade after the inducer is withdrawn. These effects take usually days to even weeks to fully manifest when concerning rapidly metabolized compounds (Tran et al. 1999). The time-dependent effects are even slower when dealing with steady-state levels of compounds with long half-lives. Thus, the outcome of adding an inducer to the patient’s established drug regimen can be difficult to detect in clinical setting if the physician is unaware of the anticipated effect. The effect of the induction is even more difficult to discern when dealing with intermittent exposures as is common with environmental toxicants as both victims and perpetrators of induction. For drugs and toxicants active in their parent form, CYP induction increases the elimination of compounds and decreases therapeutic and toxic effects, respectively. For prodrugs and toxicants that have active metabolites formed by CYP enzymes, enhanced pharmacodynamic and toxic effects could result.

The consequences of CYP induction are even more difficult to evaluate when dealing with mixtures of chemical compounds comprised of all the pharmaceutical, herbal, and environmental chemical exposures encountered by individuals in their daily lives. This is due to newly emerging findings on the combinatorial effects of chemical mixtures as activators of xenobiotic-sensing receptors. This phenomenon has been best demonstrated with PXR. It has been shown that combinations of toxic compounds such as bisphenol A analogs (Sui et al. 2012), and drugs and toxicants such as the combination of pesticide trans-nonachlor and drug 17α-ethinylestradiol (Delfosse et al. 2015), potentiate the PXR activation even at the low concentrations incapable to activate PXR by themselves. The science of the combinations is still very much a work in progress.

## Concluding remarks and lessons learnt

After intense investigation for several decades, the research field of CYP inhibition and induction has reached a rather matured stage. The basic mechanisms of both CYP inhibition and induction are now fairly well understood, although further details continue to be revealed.

The experimental tools to study CYP inhibition and induction in vitro have been well established and adopted in guidelines regulating drug development. The in vitro results can further guide the in vivo experiments. Indeed, we have moved from testing clinically commonly used individual drugs together to the rational design of studies using index drugs and reference inhibitors based on mechanistic understanding of drug–drug interactions (Tornio et al. 2019). Further development has been made in the computational tools, and the physiologically based pharmacokinetic modeling can be used to simulate in vivo conditions, extend the knowledge gained from the clinical studies, and even avoid unnecessary clinical studies (Shebley and Einolf 2019; Venkatakrishnan and Rostami-Hodjegan 2019). However, human in vivo DDI studies are still needed to definitively demonstrate the consequences of inhibition/induction, especially for the regulatory filings, and it is not likely that these studies would be deemed unnecessary in the near future.

As a result of the methodological developments, the CYP-mediated drug–drug interactions are identified early in the pharmaceutical development and no longer big surprises appear in the clinical use after approval. The early awareness of the potential CYP-mediated drug–drug interactions may also guide the drug development process to avoid strong inhibitors and inducers. Thus, especially the number of new inducers has been low among the recently approved drugs. However, there may still be unidentified inducers and inhibitors among the compounds present in our diet and various herbal remedies as well as in the environment as chemical toxicants.

The CYP-mediated interactions are now mastered rather well in the drug development process. The use of different databases and prescription aid tools has also improved application of the interaction data in the clinical practice. The widespread application of these information technology solutions is crucial as the amount of DDI data are too extensive for any individual physician to master. The progress in the pharmaceutical drug development during the recent years has resulted in design of small-molecular drugs with increasing metabolic stability. While this decreases the risk of CYP-mediated drug–drug interactions, this development may induce other types of interactions such as those mediated by various transporters (Venkatakrishnan and Rostami-Hodjegan 2019).

Although, in general, there is a good potential for prediction of the CYP inhibition and induction, unusual cases may still continue to provide surprises. For example, it was described that co-binding of two non-activating compounds to the active site of PXR may result in synergistic effect and receptor activation (Delfosse et al. 2015). This kind of cocktail effect may be possible among drugs, but perhaps more relevant in the toxicological exposure to complex mixtures. Naturally, also drugs and environmental compounds or natural substances could interact or act together. Thus, although much has been learned in the last decades regarding inhibition and induction of CYP enzymes, novel discoveries may still be made by inquiring minds.

## Data Availability

All the data are available in the text and tables of the review.

## Footnotes

1 EMA 2012, https://www.ema.europa.eu/en/documents/scientific-guideline/guideline-investigation-drug-interactions-revision-1_en.pdf; FDA 2020, https://www.fda.gov/media/134582/download and https://www.fda.gov/media/134581/download; MHLW/PMDA 2018, https://www.pmda.go.jp/files/000228122.pdf.

2 https://www.fda.gov/media/134581/download.

3 Summary of Product Characteristics, https://www.accessdata.fda.gov/drugsatfda_docs/label/2020/202992s010lbl.pdf.
